# Combating multidrug-resistant pathogens, breast cancer (MCF-7) and hepatocellular carcinoma (HepG2) using biofabricated zinc oxide nanoparticles

**DOI:** 10.1038/s41598-026-66940-0

**Published:** 2026-08-20

**Authors:** Gamal M. El-Sherbiny, Dina M. Elkhashab, M. E. Shehata, Amira Salah El-Din Youssef

**Affiliations:** 1 Botany and Microbiology Department, Faculty of Science, Al-Azhar University, Cairo, 11884 Egypt; 2 Clinical Pathology Department, National Cancer Institute, Cairo University, Cairo, Egypt; 3 Virology and Immunology Unit, Cancer Biology Department, National Cancer Institute, Cairo University, Cairo, Egypt

**Keywords:** Green synthesis, ZnO nanoparticles, Multidrug-resistant pathogens, Time–kill kinetics, Antibiofilm activity, Antioxidant activity, Breast cancer, Apoptosis, Nanotherapeutics, Biochemistry, Biotechnology, Cancer, Drug discovery, Materials science, Microbiology, Nanoscience and technology

## Abstract

The escalating global burden of multidrug-resistant (MDR) infections and cancer necessitates the development of sustainable and multifunctional therapeutic strategies. Green-synthesized zinc oxide nanoparticles (ZnONPs) have emerged as promising nanomaterials owing to their unique physicochemical properties, broad-spectrum bioactivity, and environmentally friendly production routes. In this study, ZnONPs were biosynthesized using *Citrus sinensis* (orange) peel waste extract as a renewable reducing and stabilizing agent. The biosynthesized nanoparticles were comprehensively characterized by UV–Vis spectroscopy, FTIR, XRD, DLS, zeta potential analysis, SEM, TEM, HR-TEM, and SAED. Their antimicrobial, Time–kill kinetics, antibiofilm, antioxidant, and anticancer activities were systematically evaluated against clinically relevant MDR pathogens and human cancer cell lines. The biosynthesized ZnONPs exhibited an absorption peak at 368 nm, a mean hydrodynamic size of 32.45 nm, low polydispersity (0.186), and a zeta potential of − 36.4 mV, confirming excellent colloidal stability and highly crystalline hexagonal wurtzite nanostructures. ZnONPs demonstrated broad-spectrum antimicrobial activity, with MIC and MBC/MFC values ranging from 120 to 512 and 240 to 1024 µg/mL, respectively. Time–kill assays revealed concentration- and time-dependent antimicrobial effects, achieving complete microbial eradication at 4MIC within 8–12 h, while *C. albicans* remained the least susceptible species. Moreover, ZnONPs significantly inhibited microbial biofilm formation, with inhibition rates ranging from 55.2 to 68.3% at sub-MIC concentrations. Strong antioxidant activity was observed, with DPPH and ABTS radical-scavenging IC₅₀ values of approximately 38–40 μg/mL. Importantly, ZnONPs exhibited potent, dose-dependent, and selective anticancer activity against MCF-7 and HepG2 cells, with IC₅₀ values of 58.98 and 87.93 µg/mL, respectively, while showing minimal toxicity toward normal MCF-10A cells (IC₅₀ > 250 µg/mL; selectivity index > 4.24 for MCF-7 and > 2.84 for HepG2). Confocal microscopy demonstrated preferential and concentration-dependent nanoparticle uptake by cancer cells, which closely correlated with their enhanced cytotoxicity. Mechanistic studies further confirmed apoptosis induction through activation of the intrinsic mitochondrial pathway, as evidenced by significant upregulation of the pro-apoptotic gene *BAX* (3.4-fold in MCF-7 and 2.9-fold in MDA-MB-231 cells) and marked downregulation of the anti-apoptotic gene *BCL2* (0.4-fold and 0.3-fold, respectively; *p* < 0.0001). Orange peel-mediated ZnONPs exhibit potent antimicrobial, antibiofilm, antioxidant, and selective anticancer activities, underscoring their potential as sustainable multifunctional nanotherapeutics for combating antimicrobial resistance and cancer.

## Introduction

Antimicrobial resistance (AMR) has emerged as one of the most critical global public health challenges of the twenty-first century. It is a dynamic and multifaceted phenomenon whereby bacterial pathogens develop and employ a wide range of genetic, molecular, and physiological mechanisms to withstand the inhibitory or bactericidal effects of antimicrobial agents. Although resistance is a naturally occurring evolutionary trait among microorganisms, its rapid global expansion has been largely driven by the inappropriate and excessive use of antibiotics in human medicine, veterinary practice, agriculture, and animal husbandry^1,2^. This persistent selective pressure has accelerated the emergence, evolution, and dissemination of resistant strains, significantly outpacing the discovery and development of new antimicrobial therapies and thereby compromising the effectiveness of conventional treatment strategies^3,4^.

The global burden of AMR continues to increase at an alarming rate. Current estimates suggest that, in the absence of effective mitigation measures, AMR-related infections could result in more than 10 million deaths annually by 2050 and generate cumulative economic losses exceeding USD 100 trillion worldwide^4,5^. Consequently, AMR is recognized not only as a major healthcare concern but also as a significant socioeconomic threat with far-reaching implications for global development and healthcare systems.

One of the principal factors contributing to antimicrobial treatment failure is the ability of pathogenic microorganisms to form biofilms. Biofilms are highly structured microbial communities that adhere to biotic or abiotic surfaces and are embedded within a self-produced extracellular polymeric substance (EPS) matrix. This protective matrix acts as a formidable physical and chemical barrier, restricting antibiotic diffusion and reducing the effectiveness of host immune defenses, thereby enhancing microbial survival under adverse conditions^6,7^. Furthermore, bacterial cells residing within biofilms often exhibit altered physiological states, including reduced metabolic activity and the formation of persister cells, which display remarkable tolerance to antimicrobial agents that primarily target actively growing microorganisms. Environmental stresses, nutrient gradients, and intercellular signaling within the biofilm microenvironment further promote adaptive responses that enhance microbial persistence and resistance under antimicrobial pressure^8,9^.

The complexity and diversity of these resistance mechanisms underscore the urgent need for alternative and more effective therapeutic approaches capable of overcoming conventional antimicrobial limitations. In this regard, nanotechnology has emerged as a promising platform for the development of next-generation antimicrobial interventions. Nanomaterials possess unique physicochemical properties, including high surface-area-to-volume ratios, tunable surface functionalities, and enhanced reactivity, which enable improved antimicrobial efficacy through multiple mechanisms of action. Moreover, nanoparticle-based systems offer opportunities for targeted drug delivery, controlled release, biofilm disruption, and synergistic interactions with existing antimicrobial agents, making them attractive candidates for combating multidrug-resistant pathogens and addressing the growing global AMR crisis^10^.

Nanotechnology has emerged as a transformative scientific discipline and technological platform with the potential to revolutionize numerous sectors, positioning it as a key driver of the next industrial era. Owing to the unique physicochemical properties of nanomaterials, including their high surface-area-to-volume ratio, enhanced reactivity, and tunable surface characteristics, nanotechnology has found extensive applications in healthcare, infection control, environmental remediation, industrial manufacturing, wastewater treatment, and agriculture^11^. These distinctive features have enabled the development of innovative solutions to address pressing global challenges in both environmental sustainability and human health.

In recent years, considerable attention has been directed toward the development of environmentally sustainable methods for nanoparticle synthesis, particularly for biomedical applications. Green synthesis has emerged as an attractive alternative to conventional chemical and physical fabrication techniques due to its eco-friendly, cost-effective, and non-toxic nature. This approach utilizes biological resources, including plant extracts, microorganisms, and naturally occurring biomolecules, as reducing, capping, and stabilizing agents, thereby eliminating the need for hazardous chemicals and energy-intensive processes^12^. In addition to minimizing environmental impact, green-synthesized nanoparticles often exhibit improved biocompatibility and enhanced biological functionality, making them particularly suitable for therapeutic and diagnostic applications.

Among the various nanomaterials investigated for antimicrobial purposes, metallic nanoparticles have attracted substantial interest because of their broad-spectrum activity and ability to overcome many limitations associated with conventional antibiotics. Unlike traditional antimicrobial agents that typically act on a single cellular target, nanoparticles can simultaneously interact with multiple bacterial structures and metabolic pathways, thereby reducing the probability of resistance development. Furthermore, their ease of large-scale production, prolonged stability, and tunable physicochemical properties make them promising candidates for next-generation antimicrobial therapies^13^. Numerous in vitro and in vivo studies have demonstrated the effectiveness of metallic nanoparticles against a wide range of pathogenic microorganisms, including multidrug-resistant strains^1,13^.

The antimicrobial efficacy of metal-based nanoparticles is attributed to several interconnected mechanisms of action. These include disruption of cell membrane integrity, induction of oxidative stress through excessive generation of reactive oxygen species (ROS), interference with intracellular metabolic processes, and modulation of cellular adenosine triphosphate (ATP) levels. ATP serves as the primary energy source for cellular activities; therefore, its depletion or dysregulation can severely impair bacterial growth, metabolism, and survival^14^. In addition, nanoparticles can induce membrane depolarization, alter protein function, damage nucleic acids, and compromise the structural integrity of the bacterial cell envelope. Importantly, nanoparticle-based antimicrobial systems have demonstrated the ability to overcome major bacterial defense strategies, including reduced drug permeability, active efflux mechanisms, biofilm formation, and intracellular persistence. By targeting multiple cellular components simultaneously, nanoparticles can effectively circumvent conventional resistance pathways and enhance antimicrobial efficacy against MDR pathogens^15^.

Given these considerations, the present study aimed to isolate and characterize clinically relevant multidrug-resistant bacterial strains and develop a sustainable nanotherapeutic strategy using orange peel waste as a renewable biogenic resource for the green synthesis of ZnONPs. Unlike conventional chemical synthesis methods, this approach simultaneously promotes agro-industrial waste valorization, minimizes environmental impact, and supports circular bioeconomy principles by converting agricultural waste into high-value functional nanomaterials. The key novelty of this work lies in the comprehensive evaluation of orange peel-mediated ZnONPs as multifunctional nanotherapeutics with combined antimicrobial, antibiofilm, and selective anticancer activities against clinically relevant MDR pathogens, breast cancer (MCF-7), and hepatocellular carcinoma (HepG2) cells while exhibiting low toxicity toward normal cells**.** This integrated approach bridges sustainable waste management, green nanotechnology, and biomedical applications, providing a promising platform for the development of eco-friendly antimicrobial and anticancer therapeutics.

## Materials and methods

### Collection of clinical specimens

Between October 2024 and October 2025, a total of 64 clinical specimens were collected from breast cancer patients attending the National Cancer Institute (NCI). The specimens included abscess samples, wound swabs, urine, and vaginal swabs, all obtained for microbiological investigation. Sample collection was performed following written informed consent from all participants. The study protocol was reviewed and approved by the Institutional Review Board of the National Cancer Institute, Cairo University, Egypt (IRB No.: IRB-00004025; Approval No.: CB2506-202-108-201), in full accordance with applicable ethical guidelines for research involving human subjects.

#### Identification of bacterial isolates

Clinical specimens were inoculated onto MacConkey agar and blood agar (Hi-Media, Mumbai, India) and incubated aerobically at 37 °C for 24 h. Following incubation, morphologically distinct bacterial colonies were selected and purified by repeated subculturing on the same media under identical incubation conditions to obtain pure isolates. Preliminary identification was carried out using standard conventional microbiological techniques, followed by confirmatory identification using the VITEK® automated system (bioMérieux SA, Marcy-l’Étoile, France) according to the manufacturer’s instructions.

#### Selection of MDR microbial isolates

In the present investigation, a total of 58 purified bacterial isolates were subjected to comprehensive antimicrobial susceptibility profiling against 15 antibiotics representing multiple therapeutic classes, including penicillins (oxacillin and ampicillin–sulbactam), second-generation cephalosporins (cefuroxime), third-generation cephalosporins (cefotaxime, ceftazidime, and ceftriaxone), aminoglycosides (gentamicin, amikacin, and tobramycin), fluoroquinolones (ciprofloxacin and levofloxacin), trimethoprim–sulfamethoxazole, carbapenems (imipenem and meropenem), and rifamycins (rifampin) (Oxoid, UK). Antimicrobial susceptibility testing was performed using the standardized Kirby–Bauer disk diffusion method, and inhibition zone diameters were interpreted according to the Clinical and Laboratory Standards Institute (CLSI) guidelines. Based on CLSI breakpoint criteria, isolates were classified as susceptible (S), intermediate (I), or resistant (R). Multidrug-resistant isolates were defined as strains exhibiting non-susceptibility to at least one antimicrobial agent in three or more antimicrobial classes. Notably, several isolates displayed extensive resistance phenotypes, including complete resistance to all tested antibiotics, emphasizing the critical and continuously escalating threat of antimicrobial resistance among clinically significant bacterial pathogens. Antifungal susceptibility assessment of *Candida* isolates was conducted using the agar disk diffusion assay in accordance with CLSI guideline M44-A2 (2018). Commercial antifungal disks (Oxoid, UK) containing fluconazole (10 µg), itraconazole (10 µg), voriconazole (1 µg), clotrimazole (10 µg), nystatin (100 IU), ketoconazole (10 µg), and amphotericin B (20 IU) were aseptically placed onto Mueller–Hinton agar (Oxoid™) supplemented with 2% glucose to optimize fungal growth and antifungal diffusion. Following incubation at 35 °C for 24 h, inhibition zone diameters were measured in millimeters using a calibrated ruler. All experiments were performed in three independent biological replicates, each analyzed in technical triplicate, and the resulting data were expressed as mean ± standard deviation (SD)^16–18^.

### Preparation of orange peel extract

Fresh *Citrus sinensis* (orange) procured from local markets in Cairo, Egypt, and immediately transported to the laboratory for processing. Healthy, disease-free peel were carefully selected, thoroughly rinsed twice with distilled water to eliminate surface impurities and contaminants, and subsequently cut into small pieces (~ 1 cm). Approximately 200 g of the prepared peel material was homogenized in 1000 mL of deionized water using a 2 L conical flask and blended at 1000 rpm for 3 min. The resulting homogenate was filtered through Whatman No. 1 filter paper under aseptic conditions, and the obtained aqueous extract was collected and stored at 4 °C for subsequent experimental applications^19^.

### Green synthesis of zinc oxide nanoparticles using orange peels extract

An aqueous 1 mM solution of zinc nitrate hexahydrate (50 mL; Sigma-Aldrich, USA) was prepared and mixed with 50 mL of orange peel extract (OPE) to obtain a final extract concentration of 2 µg/mL. The pH of the reaction mixture was carefully adjusted to 8.5 using an alkaline solution to optimize nanoparticle formation. The reaction mixture was subsequently incubated under dark conditions in a rotary shaker at 37 °C and 200 rpm for 24 h to facilitate the bioreduction of zinc ions and the biosynthesis of ZnONPs.

Control experiments consisting of zinc nitrate solutions without extract and uninoculated media were conducted in parallel to verify the specific role of the orange peel extract in nanoparticle synthesis. The progression of Zn^2^⁺ ion reduction was monitored periodically by withdrawing approximately 2 mL aliquots at defined time intervals and analyzing them using a UV–Visible spectrophotometer (JASCO V-560). The formation of a characteristic whitish suspension accompanied by a visible color transition in the reaction medium served as a preliminary indication of successful ZnONP synthesis.

Following biosynthesis, the nanoparticle suspension was centrifuged at 12,000 rpm for 30 min to recover the ZnONP precipitate. The resulting pellets were collected and dried in a hot-air oven at 60 °C until a constant weight was achieved, ensuring complete removal of residual moisture prior to subsequent physicochemical characterization and biological evaluation^11^.

#### Physicochemical characterization of biosynthesized ZnONPs

The biosynthesis of ZnONPs was preliminarily confirmed by ultraviolet–visible (UV–Vis) spectroscopy using a JASCO V-630 spectrophotometer (Japan) through detection of the characteristic surface plasmon resonance (SPR) absorption band. The crystalline structure, phase composition, and purity of the synthesized nanoparticles were subsequently analyzed by X-ray diffraction using a Shimadzu XRD-6000 diffractometer (Japan) equipped with Cu Kα radiation (λ = 1.5406 Å). Diffraction patterns were recorded over a 2θ range of 10–80° at a scanning rate of 2° min^−1^. The apparent crystallite size (ACS) of the ZnONPs was calculated using the Debye– Scherrer Eq. (1):

1 $$ACS=\frac{K\lambda }{\beta \mathrm{c}\mathrm{o}\mathrm{s}\theta }$$

where *K* is the Scherrer constant (0.9), *λ* represents the X-ray wavelength, *β* corresponds to the full width at half maximum (FWHM) of the diffraction peak expressed in radians, and *θ* denotes the Bragg diffraction angle^1^.

Fourier-transform infrared spectroscopy (Bruker Vertex 80 V, Germany) was employed to identify the functional groups involved in nanoparticle reduction, capping, and stabilization. Dried ZnONPs were homogenized with spectroscopic-grade potassium bromide (KBr), and spectra were recorded within the range of 4000–400 cm^−1^ at a spectral resolution of 4–8 cm^−1^.

The morphology, particle geometry, and nanoscale size distribution of the synthesized ZnONPs were investigated using transmission electron microscopy (TEM; JEOL JEM-2100, Japan) operated at an accelerating voltage of 80 kV at the Faculty of Science, Al-Azhar University. High-resolution transmission electron microscopy (HR-TEM) and selected area electron diffraction (SAED) analyses were additionally employed to further elucidate the crystalline structure and lattice characteristics of the nanoparticles. For TEM analysis, nanoparticle suspensions were deposited onto carbon-coated copper grids and air-dried under ambient conditions prior to imaging. Surface morphology and ultrastructural features were further examined by scanning electron microscopy (SEM; JEOL JSM-6510LV, Japan). Prior to SEM observation, dried nanoparticle samples were mounted onto aluminum stubs using conductive carbon tape and sputter-coated with a thin gold layer to enhance conductivity and imaging resolution.

Hydrodynamic particle size distribution, average particle diameter, and polydispersity index (PDI) were determined by DLS using a Zetasizer Nano instrument (Malvern Instruments Ltd., UK) at 25 °C. Samples were appropriately diluted with deionized water before analysis to minimize aggregation artifacts. Surface charge characteristics and colloidal stability of the synthesized ZnONPs were further evaluated by zeta potential analysis using electrophoretic light scattering measurements performed on the same instrument^1,12,13^.

### Antimicrobial activity of biosynthesized ZnONPs

The antimicrobial activity of the biosynthesized ZnONPs was systematically evaluated using the agar disk diffusion method on Mueller–Hinton agar (MHA; HiMedia Laboratories, India). The antimicrobial efficacy of ZnONPs was comparatively analyzed against orange peel extract, heat-inactivated orange peel extract, zinc nitrate hexahydrate-derived controls, and standard antimicrobial agents. The study was conducted against a panel of clinically isolated bacterial and *Candida* pathogens to assess broad-spectrum antimicrobial performance.

Microbial inocula were prepared by culturing each isolate in Mueller–Hinton broth (MHB) at 37 °C for 20 h under aerobic conditions. The resulting cultures were adjusted to a standardized turbidity corresponding to approximately 1.0 × 10⁶ CFU/mL. Subsequently, 100 µL of each standardized suspension was aseptically spread onto sterile MHA plates to generate uniform confluent microbial lawns. Sterile paper disks (6 mm diameter) were impregnated with 100 µL of each test formulation, including ZnONPs (50 µg/mL), orange peel extract, heat-inactivated extract, and zinc nitrate hexahydrate solutions at concentrations equivalent to those employed during nanoparticle biosynthesis. Commercial ciprofloxacin (10 µg) and itraconazole (10 µg) disks (Oxoid, UK) served as positive controls for antibacterial and antifungal assays, respectively, whereas untreated sterile disks were used as negative controls to exclude nonspecific inhibitory effects.

Following disk application, the inoculated plates were maintained at 4 °C for 2 h to facilitate uniform radial diffusion of the tested compounds into the agar matrix prior to incubation. The plates were subsequently incubated aerobically at 37 °C for 24 h. Antimicrobial activity was quantitatively determined by measuring the diameters of inhibition zones, including disk diameter, and expressed in millimeters (mm). All experiments were conducted in triplicate and independently repeated three times to ensure methodological accuracy, reproducibility, and analytical reliability. The obtained data were expressed as mean inhibition zone diameter ± standard deviation (SD)^3^.

#### Determine MIC of biosynthesized ZnONPs

The MICs of the biosynthesized ZnONPs and the reference antibiotic ciprofloxacin (Oxoid, UK) against MDR clinical microbial isolates were determined using the broth microdilution method in accordance with the Clinical and Laboratory Standards Institute (CLSI) guidelines (2024)^16–18^. Briefly, MDR bacterial isolates were cultured overnight in Mueller–Hinton broth (MHB) at 37 °C under continuous agitation (180 rpm) to ensure optimal bacterial growth and physiological stabilization. The resulting cultures were subsequently adjusted to a standardized inoculum density of approximately 1.0 × 10⁶ CFU/mL using turbidity-based calibration to maintain experimental uniformity and reproducibility.

MIC determinations were carried out in sterile UV-sterilized 96-well polystyrene microtiter plates (SPL Life Sciences, Korea). Aliquots of 100 µL of the standardized bacterial suspension were dispensed into each well, followed by the addition of 100 µL of two-fold serial dilutions of ZnONPs prepared in MHB to achieve final concentrations ranging from 0 to 100 µg/mL. In parallel, ciprofloxacin (Oxoid, UK) was serially diluted over a concentration range of 0–150 µg/mL and included as a reference antimicrobial agent for comparative susceptibility assessment. Appropriate experimental controls were incorporated throughout the assay, including growth control wells containing bacterial inoculum without antimicrobial agents to verify bacterial viability, and sterility control wells containing uninoculated MHB to confirm media sterility and the absence of contamination.

Following inoculation, the microtiter plates were incubated aerobically at 37 °C for 24 h under static conditions. The MIC was defined as the lowest concentration of the tested antimicrobial agent that completely inhibited visible bacterial growth, as indicated by the absence of turbidity compared with untreated growth control wells. All experiments were performed in triplicate and independently repeated to ensure analytical reliability, methodological reproducibility, and experimental accuracy.

#### Bactericidal and fungicidal kinetics of biofabricated ZnONPs

The bactericidal and fungicidal kinetics of the biosynthesized ZnONPs were evaluated against *Escherichia coli*, *Klebsiella pneumoniae*, *Staphylococcus aureus*, *Bacillus subtilis*, and *Candida albicans* using a standard time–kill assay with minor modifications^16–18^. Fresh microbial suspensions were prepared in Mueller–Hinton broth (for bacteria) or Sabouraud dextrose broth (for *C. albicans*) and adjusted to approximately 1 × 10⁶ CFU/mL.

Microbial cultures were exposed to ZnONPs at concentrations corresponding to 1/2 MIC, MIC, 2MIC, and 4MIC, while untreated cultures served as growth controls. The inoculated tubes were incubated at 37 °C under continuous shaking, and aliquots were collected at predetermined time intervals (0, 1, 2, 4, 8, 12, and 24 h). Serial ten-fold dilutions were prepared in sterile phosphate-buffered saline (PBS), plated onto Mueller–Hinton agar or Sabouraud dextrose agar, and incubated for 24–48 h to determine viable colony counts.

The antimicrobial activity of ZnONPs was expressed as log₁₀ colony-forming units per milliliter (log₁₀ CFU/mL) as a function of time. Bactericidal or fungicidal activity was defined as a reduction of ≥ 3 log₁₀ CFU/mL relative to the initial inoculum, whereas complete eradication was considered when microbial counts reached the limit of detection^16^. All experiments were performed in triplicate, and the results are presented as mean ± standard deviation (SD).

#### Antibiofilm properties of ZnONPs

Following MIC determination of biosynthesized ZnONPs and ciprofloxacin against representative MDR clinical isolates, a sub-inhibitory concentration corresponding to three-quarters of the MIC (3/4 MIC) was selected for subsequent antibiofilm assays. This sublethal concentration was deliberately employed to specifically assess the inhibitory effects of ZnONPs and ciprofloxacin on biofilm development while minimizing confounding influences arising from direct bactericidal activity or pronounced growth suppression^1^.

The antibiofilm potential of ZnONPs and ciprofloxacin was evaluated using complementary semi-quantitative methodologies, including the microtiter plate assay (MPA), according to established protocols. The standardized ¾ MIC concentration was consistently applied across all experimental conditions to systematically investigate its effects on initial bacterial adhesion, biofilm maturation, and extracellular polymeric substance (EPS) matrix formation.

To ensure experimental rigor and analytical validity, multiple control groups were incorporated, including: (i) uninoculated culture medium as a blank control, (ii) wells containing ZnONPs without bacterial inoculation to assess potential background absorbance or optical interference, and (iii) untreated bacterial cultures serving as positive controls for maximal biofilm formation. The negligible absorbance observed in all negative controls confirmed that recorded optical density values were attributable exclusively to biofilm biomass and were not influenced by the intrinsic optical properties of ZnONPs or residual phytochemical constituents.

For quantitative evaluation of bacterial viability, treated and untreated cultures were serially diluted in sterile phosphate-buffered saline (PBS) and plated onto Tryptic Soy Agar (TSA; Oxoid, England) using the standard spread plate technique. Plates were incubated aerobically at 37 °C for 24 h, after which viable colonies were enumerated and expressed as colony-forming units per milliliter (CFU/mL). Viable counts were determined both prior to and following nanoparticle exposure to accurately quantify ZnONP-mediated reductions in microbial survival. All experiments were conducted in triplicate and independently repeated to ensure reproducibility, statistical robustness, and methodological reliability.

### Antioxidant activity of biosynthesized ZnONPs

#### DPPH radical scavenging assay

The antioxidant activity of ZnONPs, orange peel extract, and zinc nitrate hexahydrate were evaluated using the 2,2-diphenyl-1-picrylhydrazyl (DPPH) radical scavenging assay, following the method described by Fareid et al.^10^. Serial dilutions of ZnONPs, orange peel extract, and zinc nitrate hexahydrate (0–100 µg/mL) were prepared, and 100 µL of each concentration was mixed with 100 µL of freshly prepared DPPH solution (0.1 mmol/L in methanol). Ascorbic acid at corresponding concentrations was used as the positive control, while methanol served as the blank.

The reaction mixtures were incubated in the dark at 27 °C for 20 min, after which the absorbance was measured at 517 nm using a UV–Vis spectrophotometer. The percentage of DPPH radical scavenging activity was calculated according to the following Eq. (2):

2 $$DPPH\,Scavenging\,Activity (\%)=\frac{{A}_{control}-{A}_{sample}}{{A}_{control}}\times 100$$

where ${A}_{control}$ represents the absorbance of the DPPH solution without antioxidant (negative control), and ${A}_{sample}$ represents the absorbance of the reaction mixture containing either ZnONPs, orange peel extract, zinc nitrate hexahydrate acid or ascorbic acid. The half-maximal inhibitory concentration (IC₅₀), defined as the concentration required to scavenge 50% of DPPH radicals, was determined by plotting the percentage scavenging activity against the logarithm of concentration and fitting the data using nonlinear regression analysis in GraphPad Prism software. All experiments were conducted in triplicate, and the results are presented as mean ± standard deviation (SD).

#### ABTS radical cation decolorization assay

The ABTS radical scavenging activity of ZnONPs, orange peel extract, zinc nitrate hexahydrate and ascorbic acid were assessed according to the method of Fareid et al.^10^, with slight modifications. The ABTS radical cation (ABTS) was generated by mixing 7 mmol/L ABTS solution with 2.4 mmol/L potassium persulfate, followed by incubation in the dark at 25 °C for 12–16 h. Prior to analysis, the resulting ABTS solution was diluted with ethanol (1:89, v/v) to obtain an absorbance of 0.70 ± 0.02 at 734 nm.

Serial dilutions of ZnONPs, orange peel extract, zinc nitrate hexahydrate and ascorbic acid (0–100 µg/mL) were prepared and mixed with the diluted ABTS⁺ solution. Methanol was used as the blank. Following incubation, absorbance was measured at 734 nm, and the percentage radical scavenging activity was calculated relative to the control using the equation described for the DPPH assay.

The IC₅₀ values were determined from concentration–response curves generated by plotting the percentage scavenging activity against the logarithm of concentration and applying nonlinear regression analysis using GraphPad Prism software. All measurements were performed in triplicate, and the results are expressed as mean ± SD from three independent experiments.

### Cytotoxicity of biosynthesized ZnONPs against MCF-7, HepG2 and MDA-MB-231 cell lines

The antiproliferative potential of biosynthesized ZnONPs was assessed against human cancer cell lines using the MTT colorimetric assay, following the method of Chandramohan et al.^20^ with slight modifications. Human breast adenocarcinoma (MCF-7), hepatocellular carcinoma (HepG2), and normal human breast epithelial (MCF-10A) cell lines were procured from the American Type Culture Collection (ATCC, Rockville, MD, USA). Cells were seeded into 96-well culture plates and incubated overnight under standard culture conditions to ensure adequate attachment and stabilization.

Following incubation, the culture medium was replaced with fresh medium supplemented with increasing concentrations of biosynthesized ZnONPs (6.25–250 µg/mL). Cells were then incubated for 24 h at 37 °C in a humidified atmosphere containing 5% CO₂. Untreated cells maintained under identical conditions served as the negative control. After treatment, cells were gently washed three times with cold phosphate-buffered saline (PBS) to eliminate residual compounds.

To evaluate cellular metabolic activity, MTT solution (0.5 mg/mL) was added to each well and incubated for 2–5 h at 37 °C, allowing viable cells to reduce the tetrazolium salt into insoluble purple formazan crystals. The supernatant was subsequently removed, and the formed crystals were dissolved in 200 µL dimethyl sulfoxide (DMSO). Absorbance was measured at 570 nm using a microplate reader, and cell viability was determined based on optical density (OD) values.

Cell viability and cytotoxicity were calculated according to the following Eqs. (3 and 4):

3 $$ {\mathrm{Cell}}\;{\mathrm{viability}}\left( \% \right) = \left( {{\mathrm{OD}}_{{{\mathrm{treated}}}} \, /{\mathrm{OD}}_{{{\mathrm{control}}}} } \right) \times {1}00 $$

4 $$ {\mathrm{Cell}}\;{\mathrm{death}}(\% ) = \, \left[ {\left( {{\mathrm{OD}}_{{{\mathrm{control}}}} - {\mathrm{OD}}_{{{\mathrm{treated}}}} } \right)/{\mathrm{OD}}_{{{\mathrm{control}}}} } \right] \times {1}00 $$

The half-maximal inhibitory concentration (IC₅₀) values were determined from dose–response curves and used to compare the cytotoxic efficacy of ZnONPs against cancerous and non-cancerous cell lines.

### Cellular uptake of biosynthesized ZnONPs by confocal laser scanning microscopy (CLSM)

Cellular uptake and intracellular localization of the biosynthesized ZnONPs were investigated using fluorescein isothiocyanate (FITC)-labeled nanoparticles combined with two-dimensional (2D) and three-dimensional (3D) confocal laser scanning microscopy. Briefly, ZnONPs were fluorescently labeled by incubation with FITC (1 mg/mL) in carbonate buffer (pH 9.0) for 2 h at room temperature in the dark. Unbound FITC was removed by repeated centrifugation (12,000×*g*, 15 min) and washing with phosphate-buffered saline (PBS) until the supernatant became colorless, yielding purified FITC-labeled ZnONPs suitable for cellular imaging^1,2^.

MCF-7, HepG2 and MCF-10A cells were seeded onto sterile glass-bottom confocal dishes at a density of 1 × 10^5^ cells/well and cultured overnight at 37 °C in a humidified atmosphere containing 5% CO₂. Cells were subsequently treated with FITC-labeled ZnONPs at final concentrations of 6.25, 12.50, 25, and 50 µg/mL for 24 h, while untreated cells served as negative controls.

Following incubation, cells were washed three times with PBS to remove non-internalized nanoparticles, fixed with 4% paraformaldehyde for 15 min, and counterstained with 4′,6-diamidino-2-phenylindole (DAPI; 1 µg/mL) for 10 min to visualize cell nuclei. Fluorescence images were acquired using a confocal laser scanning microscope equipped with 488 nm (FITC) and 405 nm (DAPI) laser lines. For qualitative assessment of intracellular nanoparticle localization, three-dimensional Z-stack images were collected at 0.3–0.5 µm optical intervals**,** and 3D reconstructions were generated using the microscope software. All imaging parameters, including laser power, detector gain, pinhole size, and exposure time, were kept constant for all experimental groups to ensure reliable comparison^1,2^.

Cellular uptake of ZnONPs was quantitatively evaluated by measuring the mean fluorescence intensity (MFI) using ImageJ software (NIH, USA) from at least 10 randomly selected microscopic fields per treatment. Fluorescence intensity was normalized to the untreated control and expressed as mean ± standard deviation (SD) from three independent experiments.

### Effects of ZnONPs on apoptosis-related gene expression

Human breast carcinoma cell lines, including MCF-7 and MDA-MB-231 were treated with biosynthesized ZnONPs at a concentration of 5 µg/mL for 24 h to investigate their effects on apoptosis-related gene regulation at the molecular level. Untreated cells were included as negative controls, whereas doxorubicin (5 µg/mL; Sigma-Aldrich, USA), a well-established chemotherapeutic agent known to induce apoptosis, was used as a positive control to validate cytotoxic and pro-apoptotic responses^21^. Following treatment, total cellular RNA was extracted using a commercially available RNA isolation kit (Thermo Fisher Scientific, USA) in accordance with the manufacturer’s standardized protocol. The concentration, integrity, and purity of the isolated RNA were evaluated spectrophotometrically using a NanoDrop™ system. Only high-quality RNA samples exhibiting an A260/A280 ratio within the range of 1.8–2.0 were selected for downstream molecular analyses. Complementary DNA (cDNA) synthesis was performed using 1 µg of total RNA via reverse transcription, primed with a combination of oligo(dT) and random hexamer primers, according to the manufacturer’s instructions. Quantitative real-time polymerase chain reaction (qRT-PCR) was subsequently conducted using SYBR Green-based fluorescence chemistry on a real-time PCR system to determine the transcriptional expression levels of the pro-apoptotic gene *BAX* and the anti-apoptotic gene *BCL2*. The housekeeping gene *GAPDH* was used as an internal endogenous control for normalization of gene expression. Primer sequences employed in the amplification reactions are presented in Table1. Thermal cycling conditions consisted of an initial denaturation step at 95 °C for 10 min, followed by 40 amplification cycles comprising denaturation at 95 °C for 15 s, annealing at 59–60 °C for 40 s, and extension at 72 °C for 20 s. Relative gene expression levels were calculated using the comparative threshold cycle method (2^−ΔΔCt^) and expressed as fold-change differences relative to untreated control cells after normalization to *GAPDH* expression^21^.

**Table 1:** Primer sequences for *BAX, BCL2*, and *GAPDH*.

Gene	Forward primer	Reverse primer
*BAX*	CGAACTGGACAGTAACATGG	CAGTTTGCTGGCAAAGTAGA
*BCL2*	GATTGTGGCCTTCTTTGAGT	ATAGGCACCCAGGGTGAT
*GAPDH*	GAAGGTGAAGGTCGGAGTCA	GAGATGGTGATGGGATTTC

### Statistical analysis

All experiments were performed in triplicate, and data are presented as mean ± SD. Statistical analyses were conducted using one-way ANOVA followed by Tukey’s post hoc test in GraphPad Prism 10.0, with *p* < 0.05 considered statistically significant.

## Results and discussion

### Isolation and identification of microbial isolates

A total of 64 clinical microbial isolates were recovered from diverse clinical specimens, including abscess aspirates, wound swabs, urine samples, and vaginal swabs. Species-level identification revealed consistently high-confidence assignments, with identification probabilities ranging from 96 to 98% across all isolates, thereby demonstrating the high discriminatory accuracy and analytical robustness of the identification platform employed.

The microbial population was predominantly composed of Gram-negative bacterial pathogens. *E. coli* was the most prevalent isolate, representing 26/64 isolates (40.56%), followed by *K. pneumoniae*, which accounted for 17/64 isolates (26.52%). Among Gram-positive bacteria, *S. aureus* was identified in 12/64 isolates (18.72%), whereas *B. subtilis* was recovered in 3/64 isolates (4.68%). The opportunistic yeast *C. albicans* constituted 6/64 isolates (9.36%). Overall, Gram-negative bacteria represented 67.08% of the total isolates, while Gram-positive bacteria and yeasts accounted for 23.40% and 9.36%, respectively (Table 2).

**Table 2:** Identification of the microbial isolates by Vitek2 system.

Microorganisms	Confidence level	% Probability	Total N. (%)
*Escherichia coli*	Excellent	98%	26 (40.56)
*Klebsiella pneumoniae*	Excellent	98%	17 (26.52)
*Staphylococcus aureus*	Excellent	98%	12 (18.72)
*Bacillus subtilis*	Excellent	98%	3 (4.68)
*Candida albicans*	Excellent	96%	6 (9.36)

The predominance of *E. coli* is consistent with its established role as a leading uropathogen and a frequent causative agent of wound and intra-abdominal infections. This reflects its natural reservoir within the gastrointestinal tract and its well-characterized ability to acquire diverse virulence and antimicrobial resistance determinants^22,23^. The substantial representation of *K. pneumoniae* aligns with its recognized clinical significance in healthcare-associated infections, particularly those involving urinary and wound sites, as well as its documented propensity for multidrug resistance and biofilm formation^24,25^. The isolation of *S. aureus* from abscess and wound specimens is likewise expected, given its extensive virulence repertoire—including adhesins, cytotoxins, and immune evasion mechanisms—that facilitate colonization and infection of skin and soft tissues^26^. Although *B. subtilis* is primarily considered an environmental organism, its recovery from clinical samples may indicate opportunistic behavior or transient contamination, particularly in the context of compromised tissue integrity or wound exposure^27^.

The detection of *C. albicans* (9.36%), particularly from vaginal swabs and urine specimens, underscores its importance as an opportunistic fungal pathogen responsible for mucosal and urinary tract infections. Its pathogenic potential is driven by key virulence traits, including morphological plasticity, enhanced adhesion capacity, and robust biofilm formation, all of which contribute to persistence and reduced antifungal susceptibility^28,29^. The coexistence of bacterial and fungal pathogens across multiple specimen types highlights the polymicrobial nature of many clinical infections and underscores the necessity of precise, high-confidence microbial identification to inform appropriate targeted antimicrobial therapy.

From an epidemiological perspective, the predominance of Gram-negative bacilli may suggest a substantial burden of antimicrobial resistance within the studied population, particularly in light of the global expansion of extended-spectrum β-lactamase (ESBL)-producing *E. coli* and carbapenem-resistant *K. pneumoniae* strains^30,31^. The consistently high-confidence identification rates (≥ 96%) further support the robustness of the diagnostic workflow and its suitability for guiding subsequent antimicrobial susceptibility testing and infection control interventions. Collectively, these findings emphasize the clinical importance of *Enterobacterales* and *S. aureus*, alongside a meaningful contribution of *C. albicans*, and reinforce the critical role of integrated microbiological diagnostics in optimizing patient management strategies.

### Susceptibility of microbial isolates to antimicrobial agents

Antimicrobial susceptibility testing of the 58 clinical bacterial isolates revealed a heterogeneous resistance profile with a clear predominance of multidrug resistance, particularly against β-lactam antibiotics as shown in Table 3. High resistance rates were observed for third-generation cephalosporins, including cefotaxime (86.2%), ceftazidime (77.58%), and ceftriaxone (62.2%), as well as cefuroxime (70.68%). Statistical analysis revealed significant differences in antimicrobial susceptibility among the tested antibiotics. High resistance was observed against cefotaxime, ceftazidime, and cefuroxime, whereas imipenem, meropenem, and amikacin showed the highest sensitivity. Data were analyzed using one-way ANOVA followed by Tukey’s post hoc test, with significance considered at *p* < 0.05. These findings strongly suggest the widespread presence of extended-spectrum β-lactamase (ESBL)-producing organisms within the studied isolates, which is consistent with global trends in Enterobacteriaceae resistance^26,30^. Fluoroquinolone resistance was also substantial, with ciprofloxacin showing 51.72% resistance and levofloxacin 20.68%, reflecting the increasing selective pressure from their extensive clinical use. Such resistance is commonly associated with mutations in DNA gyrase and topoisomerase IV genes, as well as efflux pump overexpression^32^. Similarly, trimethoprim/sulfamethoxazole exhibited a high resistance rate (77.58%), further limiting its empirical utility, particularly in urinary tract infections where it has historically been widely prescribed^33^. Among aminoglycosides, amikacin demonstrated the highest efficacy, with 87.92% sensitivity, followed by gentamicin (60.34%) and tobramycin (62.2%). The relatively preserved activity of amikacin may be attributed to its structural resilience against aminoglycoside-modifying enzymes, which commonly inactivate other agents in this class^34^. Carbapenems, including imipenem and meropenem, also retained high activity, with sensitivity rates of 86.2% and 87.92%, respectively, indicating their continued effectiveness as last-resort agents against resistant Gram-negative pathogens. However, the detection of resistance in 8.62% of isolates is clinically concerning and may indicate the emergence of carbapenemase-producing strains^35^. Oxacillin resistance was observed in 49.99% of isolates, suggesting a high prevalence of methicillin-resistant *Staphylococcus aureus* (MRSA), which poses significant therapeutic challenges due to its resistance to multiple β-lactam antibiotics^33^. Amoxicillin/sulbactam exhibited a balanced profile, with equal proportions of resistant and sensitive isolates (46.54%), reflecting partial restoration of β-lactam activity by β-lactamase inhibition, though not sufficient to overcome high-level resistance mechanisms^36^. Rifampin showed moderate activity (67.23% sensitivity), while intermediate resistance levels across several antibiotics indicate the presence of borderline susceptibility, which may contribute to treatment failure if suboptimal dosing is applied. Overall, the observed resistance patterns highlight the limited efficacy of commonly used antibiotics and underscore the necessity for routine susceptibility testing to guide targeted therapy. From a clinical perspective, the high resistance rates to cephalosporins, fluoroquinolones, and sulfonamides suggest that empirical treatment regimens relying on these agents may be inadequate in the studied setting. In contrast, aminoglycosides (particularly amikacin) and carbapenems remain the most effective therapeutic options. The emergence of resistance even to last-line agents emphasizes the urgent need for antimicrobial stewardship programs, infection control measures, and continuous surveillance to mitigate the spread of resistant pathogens.

**Table 3:** Antibiotic susceptibility profile of bacterial isolates (n = 58).

No	Antibiotics	Resistance N (%)	Intermediate N (%)	Sensitive N (%)	p-value
1	Oxacillin	29 (49.99%)	7 (12.06%)	22 (37.92%)	< 0.05*
2	Amikacin	5 (8.62%)	2 (3.44%)	51 (87.92%)	< 0.01**
3	Levofloxacin	12 (20.68%)	3 (5.1%)	43 (77.58%)	< 0.05*
4	Cefuroxime	41 (70.68%)	0(0.0%)	17 (29.30%)	< 0.001***
5	Ciprofloxacin	30 (51.72%)	5 (8.62%)	23 (39.65%)	< 0.05*
6	Ceftriaxone	40 (62.2%)	2 (3.44%)	16 (27.58%)	< 0.001***
7	Ceftazidime	43 (77.58%)	2 (3.44%)	13 (22.41%)	< 0.001***
8	Gentamicin	19 (32.75%)	4 (6.89%)	35 (60.34%)	< 0.05*
9	Cefotaxime	50 (86.2%)	0(0.0%)	8 (13.79%)	< 0.0001****
10	Imipenem	5 (8.62%)	3 (5.1%)	50 (86.2%)	< 0.01**
11	Meropenem	5 (8.62%)	2 (3.44%)	51 (87.92%)	< 0.01**
12	Amoxicillin/Sulbactam	27 (46.54%)	4 (6.89%)	27 (46.54%)	NS
13	Trimethoprim/sulfamethoxazole	43 (77.58%)	0(0.0%)	15 (25.86%)	< 0.001***
14	Rifampin	17 (29.30%)	2 (3.44%)	39 (67.23%)	< 0.05*
15	Tobramycin	14 (24.13%)	4 (6.89%)	40 (62.2%)	< 0.05*

Significance levels: *p < 0.05, **p < 0.01, ***p < 0.001, ****p < 0.0001; NS: non-significant.

The antifungal susceptibility profile demonstrated substantial variability in the inhibitory activities of the tested antifungal agents against the examined *Candida* isolates (Table 4). Among all evaluated compounds, voriconazole exhibited the highest antifungal efficacy, producing the largest inhibition zone diameter (23.0 ± 2.0 mm), followed by clotrimazole (21.0 ± 2.2 mm), amphotericin B (20.0 ± 1.7 mm), and ketoconazole (20.0 ± 2.4 mm). Statistical analysis revealed significant differences among antifungal treatments (p < 0.05). Voriconazole was assigned to Tukey group “a”, indicating significantly superior antifungal activity compared with fluconazole and itraconazole, which exhibited the lowest inhibitory effects and were grouped under “d”. The pronounced activity of voriconazole may be attributed to its broad-spectrum triazole activity and enhanced affinity for fungal cytochrome P450-dependent lanosterol 14α-demethylase, resulting in effective disruption of ergosterol biosynthesis and fungal membrane integrity^37^. The strong inhibitory activity observed for amphotericin B is consistent with its well-established fungicidal mechanism involving direct binding to membrane ergosterol, leading to pore formation, membrane destabilization, and leakage of intracellular components^38^. Similarly, clotrimazole and ketoconazole demonstrated considerable antifungal potency, which may be associated with inhibition of sterol biosynthesis and impairment of fungal membrane permeability^39^. In contrast, fluconazole and itraconazole displayed comparatively weak antifungal activity, with inhibition zone diameters of 12.2 ± 1.2 mm and 12.0 ± 1.0 mm, respectively. The reduced susceptibility toward these azole derivatives may reflect the emergence of adaptive resistance mechanisms, including overexpression of efflux pumps, mutations in ERG11 encoding lanosterol demethylase, alterations in membrane sterol composition, and biofilm-associated resistance phenotypes^40,41^. These findings are particularly relevant for opportunistic fungal pathogens such as *C. albicans*, where prolonged azole exposure frequently contributes to multidrug resistance and therapeutic failure^42^.

**Table 4:** In vitro antifungal activity against *Candida* isolates based on inhibition zone diameters (mm, Mean ± SD, n = 6).

Antifungal agent	Inhibition zone diameter (mm), Mean ± SD	Range (mm)	Tukey group*
Fluconazole	12.2 ± 1.2	8–20	d
Voriconazole	23.0 ± 2.0	16–28	a
Amphotericin B	20.0 ± 1.7	18–31	b
Itraconazole	12.0 ± 1.0	0–19	d
Ketoconazole	20.0 ± 2.4	12–28	b
Nystatin	17.0 ± 1.8	11–23	c
Clotrimazole	21.0 ± 2.2	12–27	ab

*Different letters indicate statistically significant differences between antifungal agents according to one-way ANOVA followed by Tukey’s post hoc test (p < 0.05).

The relatively broad inhibition ranges observed among several antifungal agents further suggest variability in strain-dependent susceptibility patterns. Such heterogeneity may arise from genetic diversity, differences in cell wall architecture, biofilm-forming capacity, and differential expression of resistance determinants among fungal isolates^43^. The statistically significant superiority of voriconazole and clotrimazole compared with fluconazole and itraconazole highlights the clinical importance of selecting potent antifungal agents based on susceptibility testing to optimize therapeutic outcomes and minimize the development of antifungal resistance.

### Characterization of biosynthesized ZnONPs

**Fig. 1:**
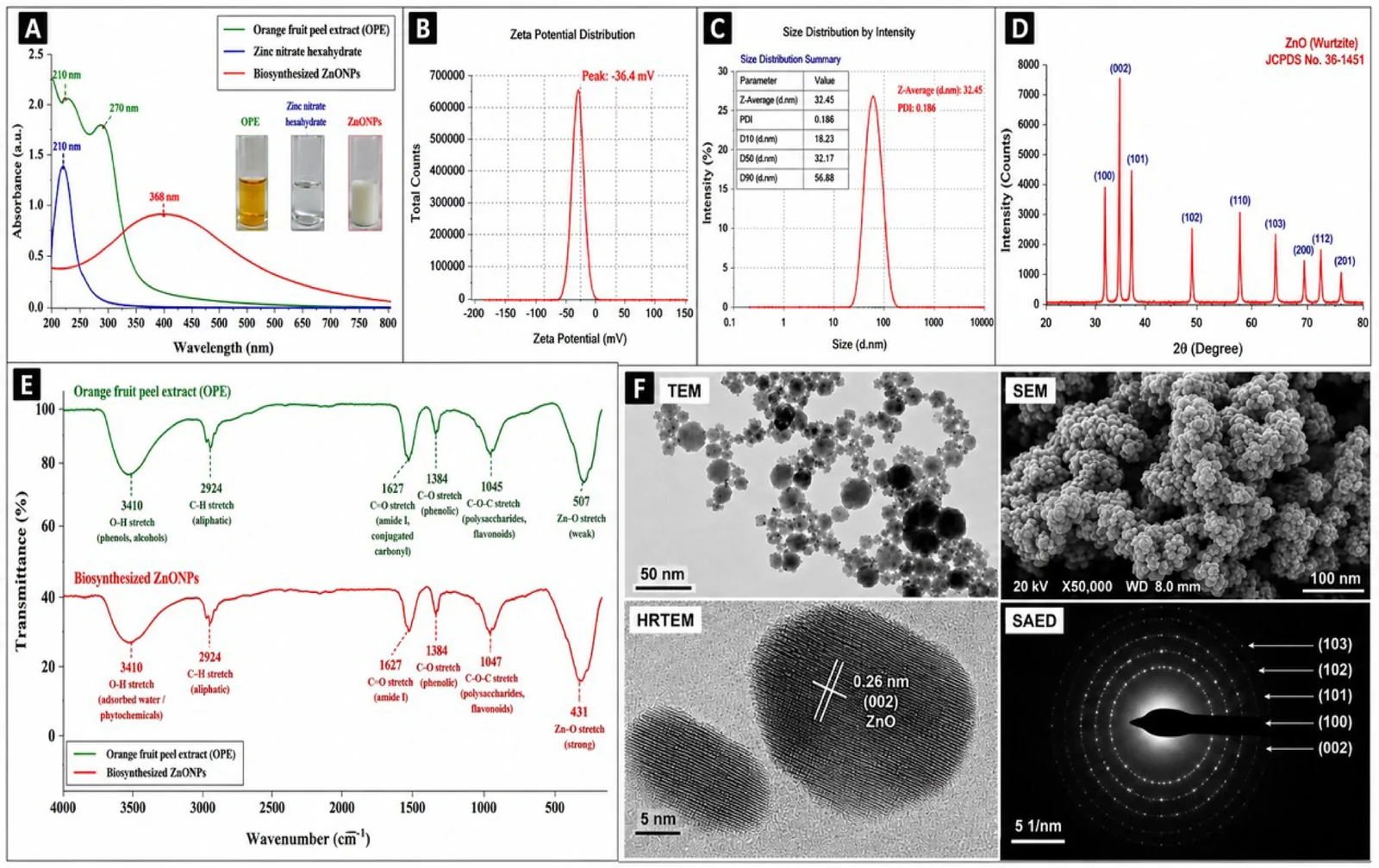
Physicochemical characterization of orange peel-mediated biosynthesized ZnONPs. (**A**) UV–Vis spectra. (**B**) Zeta potential. (**C**) DLS particle size distribution. (**D**) XRD pattern confirming the hexagonal wurtzite ZnO phase. (**E**) FTIR spectra of orange peel extract (OPE) and ZnONPs. (**F**) TEM, SEM, HR-TEM, and SAED images showing nanoscale morphology, crystallinity, lattice fringes, and phase purity of the biosynthesized ZnONPs.

The comprehensive physicochemical characterization shown in Fig. 1 (A–F) confirms the successful green synthesis of well-dispersed and highly crystalline ZnONPs with controlled size, morphology, and surface properties. UV–Vis analysis (Fig. 1A) revealed characteristic absorption bands of orange peel extract (OPE) at 229 and 270 nm, corresponding to phytochemicals such as polyphenols and flavonoids that act as reducing and stabilizing agents^44,45^. In contrast, the biosynthesized ZnONPs exhibited a broad absorption peak at 368 nm, characteristic of ZnO excitonic transitions, confirming nanoparticle formation^46,47^. This absorption maximum agrees with previous reports on green-synthesized ZnONPs (350–380 nm)^48^. Moreover, the slight peak broadening suggests nanoscale particle distribution and strong interactions between ZnONPs and phytochemical capping molecules, which enhance colloidal stability and biological performance^49–51^. Overall, these findings demonstrate the crucial role of citrus-derived phytochemicals in the reduction, stabilization, and functionalization of ZnONPs.

Figure 1B showed that the biosynthesized ZnONPs possessed a strong negative zeta potential, indicating the presence of negatively charged phytochemical moieties adsorbed onto the nanoparticle surface during the green synthesis process. Plant-derived metabolites such as phenolics, flavonoids, proteins, tannins, terpenoids, and carboxyl-containing biomolecules commonly act as reducing and capping agents during biosynthesis, thereby contributing to nanoparticle stabilization through steric and electrostatic mechanisms^45^. The adsorption of these biomolecules onto the ZnONP surface likely generated a stable electrical double layer, which enhanced colloidal dispersion and minimized particle aggregation in aqueous media. The narrow zeta potential distribution further indicates that the synthesized ZnONPs possessed a relatively monodispersed nature with homogeneous surface characteristics. Such uniformity is highly desirable because particle aggregation significantly influences nanoparticle reactivity, bioavailability, catalytic efficiency, and antimicrobial performance. The high magnitude negative surface charge observed in this study is therefore indicative of excellent suspension stability and suggests that the ZnONPs may retain their nanoscale properties over extended storage periods^50,51^

From a mechanistic perspective, the electrostatic repulsion generated between similarly charged nanoparticles reduces van der Waals attractive interactions, thereby preventing coagulation and sedimentation. Previous studies have similarly reported that green-synthesized ZnONPs exhibiting zeta potential values between − 25 and − 40 mV possess enhanced colloidal stability and improved biological performance^45,46^. The present finding of − 36.4 mV therefore confirms the successful surface functionalization and stabilization of ZnONPs through biological synthesis routes. Moreover, surface charge plays a crucial role in determining nanoparticle interactions with microbial cell membranes and biological systems. Negatively charged ZnONPs may exhibit improved dispersion and greater accessibility to target cells, enhancing ROS generation and antimicrobial activity. The stable colloidal nature observed in the present study may therefore contribute positively to the biomedical, catalytic, antioxidant, and antimicrobial applications of the synthesized ZnONPs^52^.

Dynamic light scattering (Fig. 1C) revealed a hydrodynamic diameter of 32.45 nm and a low polydispersity index (PDI = 0.186), indicating a homogeneous and highly dispersed nanoparticle population with minimal aggregation. The narrow, monomodal size distribution confirms the effective stabilization of ZnONPs by phytochemicals present in the orange peel extract. The hydrodynamic diameter measured by DLS is larger than the particle size observed by TEM or SEM because it includes the hydration layer and adsorbed biomolecules surrounding the ZnO core^53–55^. These results agree with previous studies reporting hydrodynamic sizes of 20–60 nm and low PDI values for green-synthesized ZnONPs^52,56^, confirming the successful production of stable nanoparticles with desirable physicochemical properties for biomedical and environmental applications.

The XRD analysis of the biosynthesized ZnONPs confirmed the successful formation of highly crystalline ZnO nanostructures with a characteristic hexagonal wurtzite crystal phase (Fig. 1D and Table 5). The diffraction pattern exhibited several intense and well-defined Bragg reflection peaks at 2θ values typically corresponding to the crystallographic planes of ZnO, including (100), (002), (101), (102), (110), (103), and (112). These diffraction peaks are in good agreement with the standard Joint Committee on Powder Diffraction Standards (JCPDS) card No. 36–1451 for hexagonal ZnO, confirming the phase purity and crystalline nature of the synthesized nanoparticles^57^. The sharp and narrow diffraction peaks observed in the XRD spectrum indicate a high degree of crystallinity and the formation of well-ordered nanoscale ZnO crystals. The absence of additional impurity peaks further demonstrates that the biosynthesis process effectively produced pure ZnONPs without detectable secondary phases such as zinc hydroxide or other zinc-containing intermediates. This finding highlights the efficiency of the biological reducing and stabilizing agents involved in directing nanoparticle nucleation and crystal growth during synthesis.

**Table 5:** XRD peak parameters of biosynthesized ZnONPs.

No	2θ (°)	d-spacing (Å)	Intensity (Counts)	FWHM (°)	hkl
1	31.72	2.819	2475	0.18	(100)
2	34.42	2.603	7321	0.16	(002)
3	36.25	2.475	3864	0.17	(101)
4	47.54	1.912	1658	0.2	(102)
5	56.6	1.624	2421	0.22	(110)
6	62.85	1.479	1428	0.23	(103)
7	66.38	1.405	896	0.24	(200)
8	68.03	1.379	1123	0.25	(112)
9	72.54	1.304	721	0.26	(201)

The strong diffraction intensity associated with the (101) plane suggests preferential crystal orientation and enhanced structural stability of the synthesized ZnONPs. In ZnO nanostructures, the (101) reflection is commonly reported as the most thermodynamically stable plane and is frequently associated with improved optical, catalytic, and antimicrobial properties^57,58^. The observed crystalline characteristics therefore indicate the successful synthesis of structurally stable ZnONPs suitable for multifunctional nanobiotechnological applications. The average apparent crystallite size (ACS) of the biosynthesized ZnONPs was calculated using the Debye–Scherrer Eq. (1), yielding an average crystallite size of approximately 47 nm, confirming the successful formation of nanocrystalline ZnO. The nanosized crystallite dimensions contribute significantly to the enhanced surface area, increased active surface sites, and improved physicochemical reactivity of ZnONPs. The crystalline structure observed in the present study is particularly important because the physicochemical and biological activities of ZnONPs are strongly influenced by crystallinity, crystal defects, and particle size. Highly crystalline ZnONPs possess superior electron mobility, photocatalytic efficiency, and reactive oxygen species (ROS) generation capacity compared with amorphous structures^58^. Consequently, the well-defined crystalline architecture of the biosynthesized ZnONPs may enhance their antimicrobial, antioxidant, photocatalytic, and anticancer activities. Furthermore, the biosynthetic approach likely facilitated controlled crystal growth through the interaction of phytochemicals present in the biological extract. Biomolecules such as flavonoids, polyphenols, proteins, and terpenoids can bind selectively to crystal facets during nanoparticle formation, thereby influencing crystal morphology, growth kinetics, and stabilization^13^. The formation of highly crystalline ZnONPs in this study therefore demonstrates the effectiveness of green synthesis routes in producing structurally stable and phase-pure nanomaterials. Previous studies on plant-mediated ZnONP synthesis have similarly reported XRD diffraction patterns characteristic of hexagonal wurtzite ZnO with strong crystalline reflections and average crystallite sizes ranging from 15 to 50 nm^46,56^ The present findings are therefore consistent with earlier reports and further validate the successful biosynthesis of crystalline ZnONPs through environmentally friendly biological methods. Overall, the XRD analysis conclusively demonstrates that the biosynthesized ZnONPs possess a highly crystalline hexagonal wurtzite structure with excellent phase purity and nanoscale crystallite dimensions. These structural characteristics strongly support the suitability of the synthesized ZnONPs for advanced biomedical, catalytic, environmental, and pharmaceutical applications.

FTIR analysis (Fig. 1E) confirmed the key role of orange peel phytochemicals in the reduction and stabilization of ZnONPs. Characteristic bands at 3410, 2924, 1627, 1384, and 1045 cm^−1^ corresponded to hydroxyl, aliphatic, carbonyl, and C–O functional groups associated with phenolics, flavonoids, proteins, and polysaccharides^1,46^. Following nanoparticle synthesis, shifts in peak positions and intensities indicated strong interactions between Zn^2^⁺ ions and these biomolecules, which remained adsorbed on the nanoparticle surface as natural capping agents. Notably, the appearance of a distinct band at 431 cm^−1^, assigned to Zn–O stretching vibrations, confirmed the successful formation of crystalline ZnONPs^59^. These findings demonstrate that orange peel waste serves as an effective and sustainable source of reducing and stabilizing agents for the green synthesis of functional ZnONPs with enhanced colloidal stability and biological activity.

The biosynthesized ZnO nanoparticles were comprehensively characterized by TEM, SEM, HR-TEM, and selected-area electron diffraction (SAED), confirming their nanoscale morphology, crystallinity, and phase purity (Fig. 1F). TEM images revealed predominantly quasi-spherical nanoparticles with slight agglomeration, likely resulting from their high surface energy and strong interparticle interactions. The particle sizes ranged from approximately 20 to 50 nm, with an average diameter of ~ 35 ± 10 nm, which is in close agreement with the average crystallite size (~ 47 nm) estimated from XRD using the Debye–Scherrer equation. This correspondence suggests that the biosynthesized ZnONPs are mainly single-crystalline, with minimal aggregation into larger polycrystalline structures. The small discrepancy between the TEM and XRD measurements is expected because XRD determines the size of coherent crystalline domains, whereas TEM measures the overall particle dimensions. Collectively, these findings confirm the successful synthesis of well-defined, highly crystalline ZnONPs with uniform nanoscale dimensions, highlighting their potential for biomedical and antimicrobial applications. Similar spherical or near-spherical ZnO morphologies have been widely reported in biologically mediated syntheses, where phytochemicals act as both reducing and stabilizing agents, limiting excessive growth and controlling nucleation processes^58^. SEM micrographs further supported the TEM observations, showing clustered and flower-like aggregated structures. Such hierarchical assemblies are frequently observed in biosynthesized ZnO systems and are often influenced by capping biomolecules that promote oriented attachment and secondary aggregation during nanoparticle formation^60^. These morphological features can enhance surface reactivity, which is beneficial for catalytic and antimicrobial applications.

HR-TEM images provided clear lattice fringes, indicating the high crystallinity of the synthesized ZnONPs. The measured interplanar spacing of approximately 0.26 nm corresponds to the (002) crystallographic plane of hexagonal wurtzite ZnO, confirming the formation of well-ordered crystalline domains^61^. The presence of distinct lattice fringes further indicates minimal structural defects and efficient crystallization under the employed biosynthetic conditions. The SAED pattern exhibited well-defined concentric diffraction rings indexed to the (100), (002), (101), (102), and (103) planes, confirming the polycrystalline nature of the ZnONPs with a hexagonal wurtzite structure. This diffraction behavior is consistent with standard JCPDS data for ZnO and agrees with previously reported biosynthesized ZnO nanoparticles^60^.

### Antimicrobial activity and MIC values of biosynthesized ZnONPs

**Fig. 2:**
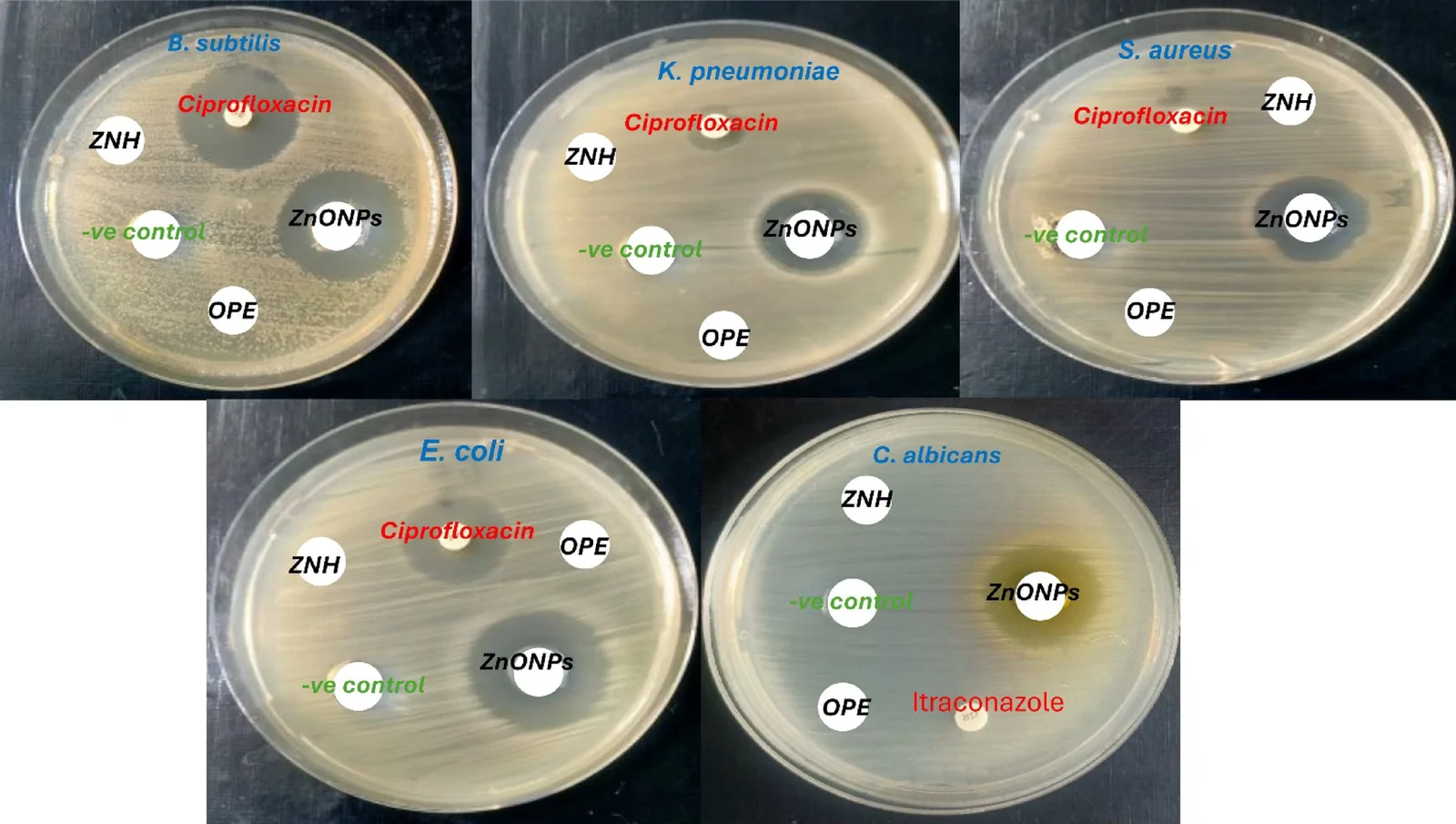
Antimicrobial activity of biosynthesized ZnONPs against Gram-positive bacteria (*B. subtilis* and *S. aureus*), Gram-negative bacteria (*E. coli* and *K. pneumoniae*), and *C. albicans* using the agar diffusion assay. OPE = orange peel extract and ZNH = zinc nitrate hexahydrate.

The antimicrobial activity of the biosynthesized ZnONPs against representative microbial isolates, including Gram-positive bacteria (*B. subtilis* and *S. aureus*), Gram-negative bacteria (*E. coli* and *K. pneumoniae*), and the fungal pathogen *C. albicans*, is summarized in Table 6 and Fig. 2. The results demonstrated that ZnONPs exhibited potent broad-spectrum antimicrobial activity against all tested microorganisms, with inhibition zone diameters ranging from 18.0 ± 1.0 to 24.0 ± 1.4 mm. No inhibitory activity was observed in the negative control, orange peel extract and zinc nitrate hexahydrate groups, confirming that the antimicrobial effects were exclusively associated with ZnONP treatment. Among the tested microorganisms, *E. coli* exhibited the highest susceptibility toward ZnONPs, producing the largest inhibition zone (24.0 ± 1.4 mm), followed by *Bacillus subtilis* (22.0 ± 1.2 mm), *K. pneumoniae* (21.0 ± 1.3 mm), *S. aureus* (19.0 ± 1.1 mm), and *C. albicans* (18.0 ± 1.0 mm). Interestingly, ZnONPs demonstrated greater inhibitory activity than ciprofloxacin against *K. pneumoniae* and *S. aureus*, where the antibiotic control showed limited or no detectable inhibition under the tested conditions. Similarly, itraconazole exhibited no measurable inhibitory activity against *C. albicans*, whereas ZnONPs retained considerable antifungal efficacy. Statistical analysis revealed significant differences among the treatment groups (p < 0.05). The Tukey grouping analysis confirmed that *E. coli* and *B. subtilis* exhibited significantly higher susceptibility to ZnONPs compared with *S. aureus* and *C. albicans*. These findings demonstrate the strong antimicrobial potential of biosynthesized ZnONPs against both bacterial and fungal pathogens, including strains exhibiting reduced susceptibility to conventional antimicrobial agents.

**Table 6:** Antimicrobial activity of biosynthesized ZnONPs against selected bacterial and fungal pathogens.

Microorganism	Orange peel extract	Zinc nitrate hexahydrate	Inhibition zone of positive control (mm)	ZnONPs inhibition zone (mm)	Negative control	Tukey group**
*B. subtilis*	0.0	0.0	20.0 ± 1.5	22.0 ± 1.2	0.0	b, a
*K. pneumoniae*	0.0	0.0	6.0 ± 1.0	21.0 ± 1.3	0.0	c, a
*S. aureus*	0.0	0.0	0.0	19.0 ± 1.1	0.0	d, b
*E. coli*	0.0	0.0	20.0 ± 1.2	24.0 ± 1.4	0.0	b, a
*C. albicans*	0.0	0.0	0.0	18.0 ± 1.0	0.0	d, b

Values are presented as mean ± SD (n = 3). Different lowercase letters indicate significant differences according to one-way ANOVA followed by Tukey’s post hoc test (p < 0.05). Ciprofloxacin and itraconazole served as positive controls.

The antimicrobial activity of the biosynthesized ZnONPs against representative bacterial and fungal pathogens is summarized in Table 7. ZnONPs exhibited broad-spectrum antimicrobial efficacy, with MIC values ranging from 120 to 512 µg/mL and corresponding MBC/MFC values ranging from 240 to 1024 µg/mL. Among the tested microorganisms, *Escherichia coli* showed the highest susceptibility, exhibiting the lowest MIC and MBC values (120 and 240 µg/mL, respectively), followed by *Bacillus subtilis* (128 and 256 µg/mL). In contrast, *Candida albicans* displayed the lowest sensitivity, with MIC and MFC values of 512 and 1024 µg/mL, respectively. Moderate antimicrobial activity was observed against *Staphylococcus aureus* and *Klebsiella pneumoniae*, which exhibited MIC/MBC values of 256/512 and 384/768 µg/mL, respectively.

**Table 7:** MIC and MBC/MFC values of biosynthesized ZnONPs and ciprofloxacin against selected microbial pathogens.

Microorganism	ZnONPs MIC (µg/mL)	MBC/MFC (µg/mL)	Ciprofloxacin MIC (µg/mL)	p-value
*Escherichia coli*	120	240	25	< 0.0001
*Klebsiella pneumoniae*	384	768	50	< 0.0001
*Staphylococcus aureus*	256	512	25	< 0.0001
*Bacillus subtilis*	128	256	25	< 0.0001
*Candida albicans*	512	1024	––	< 0.0001

****p < 0.0001.

For all tested microorganisms, the bactericidal or fungicidal concentrations were approximately twofold higher than the corresponding MIC values, indicating that higher ZnONP concentrations were required to achieve complete microbial eradication. Ciprofloxacin demonstrated superior antibacterial activity, with MIC values ranging from 25 to 50 µg/mL against bacterial isolates, whereas no antifungal activity was observed against *C. albicans*. Statistical analysis revealed highly significant differences among the tested microorganisms (*p* < 0.0001). Tukey’s post hoc analysis further confirmed significantly lower MIC and MBC values for *E. coli* and *B. subtilis* compared with *K. pneumoniae* and *C. albicans*, highlighting the differential susceptibility of Gram-positive, Gram-negative, and fungal pathogens to the biosynthesized ZnONPs.

The potent antimicrobial activity of ZnONPs may be attributed to their nanoscale dimensions, high surface-area-to-volume ratio, and physicochemical reactivity, which collectively facilitate strong interactions with microbial cell membranes^58^. ZnONPs are known to induce oxidative stress through excessive generation of ROS, including hydroxyl radicals, superoxide anions, and hydrogen peroxide, ultimately causing lipid peroxidation, protein denaturation, DNA damage, and membrane disruption^11,13^. Additionally, the gradual release of Zn^2^⁺ ions may interfere with intracellular enzymatic pathways and metabolic activities, leading to microbial cell death^11^. The relatively high susceptibility of Gram-negative bacteria, particularly *E. coli* and *K. pneumoniae*, may be associated with enhanced nanoparticle penetration through the thinner peptidoglycan layer and destabilization of the outer membrane structure^13^. Conversely, the comparatively lower susceptibility observed for *S. aureus* may be related to the thicker peptidoglycan architecture characteristic of Gram-positive bacteria, which can partially limit nanoparticle diffusion and cellular penetration^13,46^. The antifungal activity demonstrated against *C. albicans* may result from ZnONP-mediated disruption of ergosterol biosynthesis, mitochondrial dysfunction, oxidative stress induction, and inhibition of fungal biofilm formation^29^. Furthermore, phytochemical constituents derived from orange fruit peel extract likely contributed to nanoparticle stabilization and enhanced biological activity. Polyphenols, flavonoids, and phenolic acids adsorbed onto the nanoparticle surface may synergistically augment antimicrobial activity through additional membrane-disruptive and antioxidant-mediated mechanisms^62^. The strong antimicrobial performance of the biosynthesized ZnONPs highlights their potential as eco-friendly nanotherapeutic agents for combating multidrug-resistant bacterial and fungal pathogens.

#### Bactericidal and fungicidal kinetics of green-synthesized ZnONPs

**Fig. 3:**
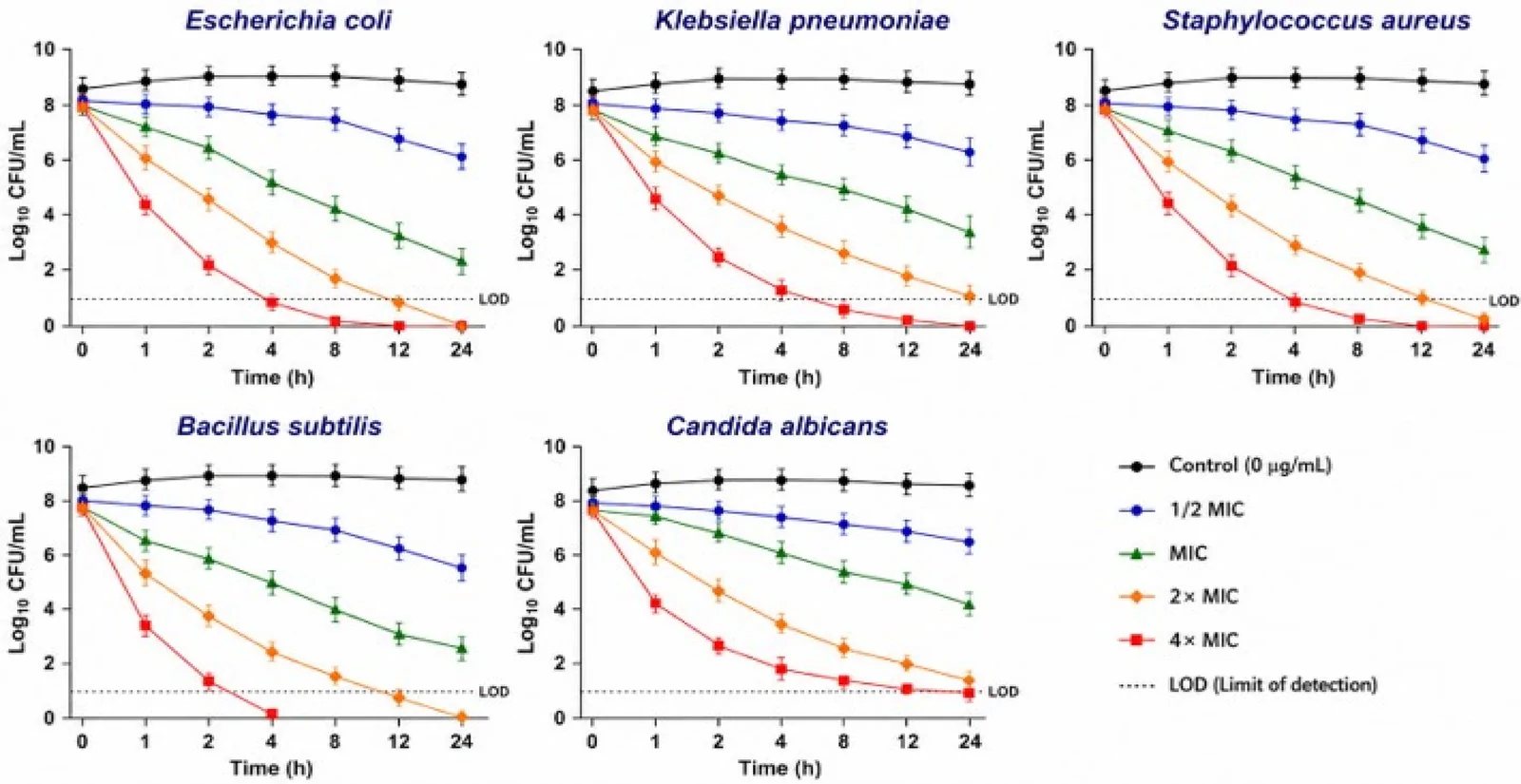
Time–kill kinetics of biosynthesized ZnONPs against *Escherichia coli*, *Klebsiella pneumoniae*, *Staphylococcus aureus*, *Bacillus subtilis*, and *Candida albicans* at ½MIC, MIC, 2MIC, and 4MIC. ZnONPs exhibited concentration- and time-dependent antimicrobial activity, with maximal bactericidal/fungicidal effects observed at 4 × MIC. Data are presented as mean ± SD (*n* = 3). LOD, limit of detection

The time–kill kinetics assay demonstrated that the biosynthesized ZnONPs exerted potent concentration- and time-dependent antimicrobial activity against *Escherichia coli*, *Klebsiella pneumoniae*, *Staphylococcus aureus*, *Bacillus subtilis*, and *Candida albicans* (Fig. 3). In all tested microorganisms, treatment with subinhibitory concentrations (½MIC) caused only a modest reduction in viable cell counts over 24 h, whereas exposure to MIC, 2MIC, and 4MIC produced progressively greater antimicrobial effects.

Among the tested strains, *E. coli* and *B. subtilis* exhibited the highest susceptibility, consistent with their lower MIC values (120 and 128 µg/mL, respectively). At 4MIC, both microorganisms showed a rapid decline in viability, reaching the limit of detection within 8–12 h. In contrast, *K. pneumoniae* and *S. aureus* displayed moderate susceptibility, requiring longer exposure times and higher ZnONP concentrations to achieve complete bacterial eradication. *Candida albicans* was the least susceptible microorganism, showing a gradual reduction in fungal burden and incomplete killing even after 24 h at MIC concentrations, in agreement with its higher MIC and MFC values.

The superior antimicrobial activity observed at 2MIC and 4MIC confirms the bactericidal and fungicidal properties of ZnONPs and suggests that their efficacy depends on both nanoparticle concentration and exposure duration. Similar concentration-dependent killing kinetics have been reported for green-synthesized ZnONPs prepared from plant extracts, which exhibited enhanced activity against Gram-positive and Gram-negative bacteria through multiple antimicrobial mechanisms^1,13^.

The antimicrobial effects of ZnONPs are primarily attributed to their nanoscale dimensions, large surface area, and ability to generate ROS, including hydroxyl radicals, superoxide anions, and hydrogen peroxide^13^. Excessive ROS production disrupts membrane integrity, induces lipid peroxidation, damages proteins and nucleic acids, and ultimately leads to microbial cell death^20^. Furthermore, intracellular release of Zn^2^⁺ ions contributes to oxidative stress and interferes with essential metabolic pathways^13,58^.

Differences in susceptibility among the tested microorganisms are likely related to variations in cell envelope architecture. The rapid killing observed for *E. coli* and *B. subtilis* may result from enhanced nanoparticle interaction with bacterial membranes, whereas the outer membrane of *K. pneumoniae* and the thick chitin–glucan cell wall of *C. albicans* provide additional barriers that reduce nanoparticle penetration and delay antimicrobial action^46^. In addition, phytochemicals originating from orange peel extract, including flavonoids, phenolic acids, and terpenoids, may remain adsorbed on the ZnONP surface and synergistically enhance microbial membrane disruption and oxidative damage^19^.

Overall, the time–kill curves confirm that orange peel-mediated ZnONPs possess broad-spectrum and sustained antimicrobial activity, with particularly strong bactericidal effects against *E. coli* and *B. subtilis*. The combination of rapid killing kinetics, multifunctional mechanisms of action, and eco-friendly synthesis highlights the potential of these nanomaterials for antimicrobial and biomedical applications.

### Antibiofilm activity of biosynthesized ZnONPs

The biofilm inhibition activity of ZnONPs and ciprofloxacin at ¾ MIC against selected microorganisms is presented in Table 8. Statistical analysis revealed significant differences among the treatment groups (p < 0.05). The use of different superscript letters (b and c) indicates that the biofilm inhibition produced by ZnONPs and ciprofloxacin differed significantly for each tested microorganism.

**Table 8:** Biofilm inhibition (%) of test microorganisms treated with ZnONPs and ciprofloxacin.

Test microorganism	ZnONPs (%)	Ciprofloxacin (%)	Statistical significance
*Escherichia coli*	55.2 ± 1.8ᵇ	78.6 ± 1.6ᶜ	p < 0.05
*Klebsiella pneumoniae*	61.4 ± 1.9ᵇ	82.3 ± 1.7ᶜ	p < 0.05
*Staphylococcus aureus*	65.7 ± 1.6ᵇ	85.9 ± 1.5ᶜ	p < 0.05
*Bacillus subtilis*	68.3 ± 1.7ᵇ	89.1 ± 1.4ᶜ	p < 0.05
*Candida albicans*	58.9 ± 1.8ᵇ	81.2 ± 1.6ᶜ	p < 0.05

Values are expressed as mean ± SD. Different superscript letters within a row indicate statistically significant differences between treatments at p < 0.05.

Ciprofloxacin exhibited significantly greater biofilm inhibition than ZnONPs against all tested microorganisms (p < 0.05). The highest biofilm inhibition was observed in *Bacillus subtilis*, with values of 68.3 ± 1.7% and 89.1 ± 1.4% for ZnONPs and ciprofloxacin, respectively. Similarly, *Staphylococcus aureus* showed high susceptibility to both treatments, recording inhibition values of 65.7 ± 1.6% for ZnONPs and 85.9 ± 1.5% for ciprofloxacin. In contrast, *Escherichia coli* exhibited the lowest inhibition percentages among the bacterial isolates, with 55.2 ± 1.8% and 78.6 ± 1.6% inhibition following treatment with ZnONPs and ciprofloxacin, respectively.

For *Klebsiella pneumoniae*, biofilm inhibition reached 61.4 ± 1.9% with ZnONPs and 82.3 ± 1.7% with ciprofloxacin, while *Candida albicans* showed inhibition values of 58.9 ± 1.8% and 81.2 ± 1.6%, respectively. The consistent increase in inhibition percentages observed with ciprofloxacin compared to ZnONPs demonstrates its superior antibiofilm efficacy under the tested conditions.

One-way analysis of variance (ANOVA) followed by a post hoc multiple-comparison test indicated that all treatment groups were significantly different from one another (p < 0.05). The low standard deviations observed across all treatments suggest good reproducibility and reliability of the experimental data. These findings confirm that both ZnONPs and ciprofloxacin possess significant antibiofilm activity, although ciprofloxacin remains the more effective treatment against the tested bacterial and fungal biofilms.

The substantial antibiofilm activity of ZnONPs may be attributed to their nanoscale dimensions, large surface-area-to-volume ratio, and enhanced physicochemical reactivity, enabling efficient interaction with microbial biofilm matrices and cellular surfaces^58^. ZnONPs are capable of disrupting early microbial adhesion, inhibiting extracellular polymeric substance (EPS) production, destabilizing mature biofilm architecture, and impairing quorum-sensing signaling pathways essential for biofilm development^11^. Furthermore, ZnONPs induce excessive reactive oxygen species (ROS) generation, leading to oxidative stress, membrane damage, protein oxidation, and microbial cell death within biofilm communities^13^. The comparatively higher antibiofilm activity observed against *B. subtilis* and *S. aureus* may be associated with the susceptibility of Gram-positive biofilm matrices to oxidative disruption and nanoparticle penetration^24^. In contrast, the relatively lower inhibition observed for *E. coli* and *K. pneumoniae* may reflect the protective role of the Gram-negative outer membrane and extracellular polysaccharide barriers that reduce nanoparticle diffusion into biofilm structures^35^. The moderate inhibition of *Candida albicans* biofilms suggests that ZnONPs can interfere with fungal adhesion and hyphal development, which are critical virulence determinants in candidiasis^29^. Additionally, phytochemical constituents derived from orange fruit peel extract likely contributed to enhanced antibiofilm efficacy through synergistic interactions with ZnONPs. Surface-bound flavonoids, phenolics, and polyphenolic compounds may enhance nanoparticle stability and further disrupt microbial membrane integrity and EPS biosynthesis^62^. Collectively, these findings demonstrate that biosynthesized ZnONPs possess significant antibiofilm potential and may serve as promising eco-friendly nanotherapeutic agents for controlling biofilm-associated multidrug-resistant microbial infections.

### Antioxidant activity of biosynthesized ZnO nanoparticles

**Fig. 4:**
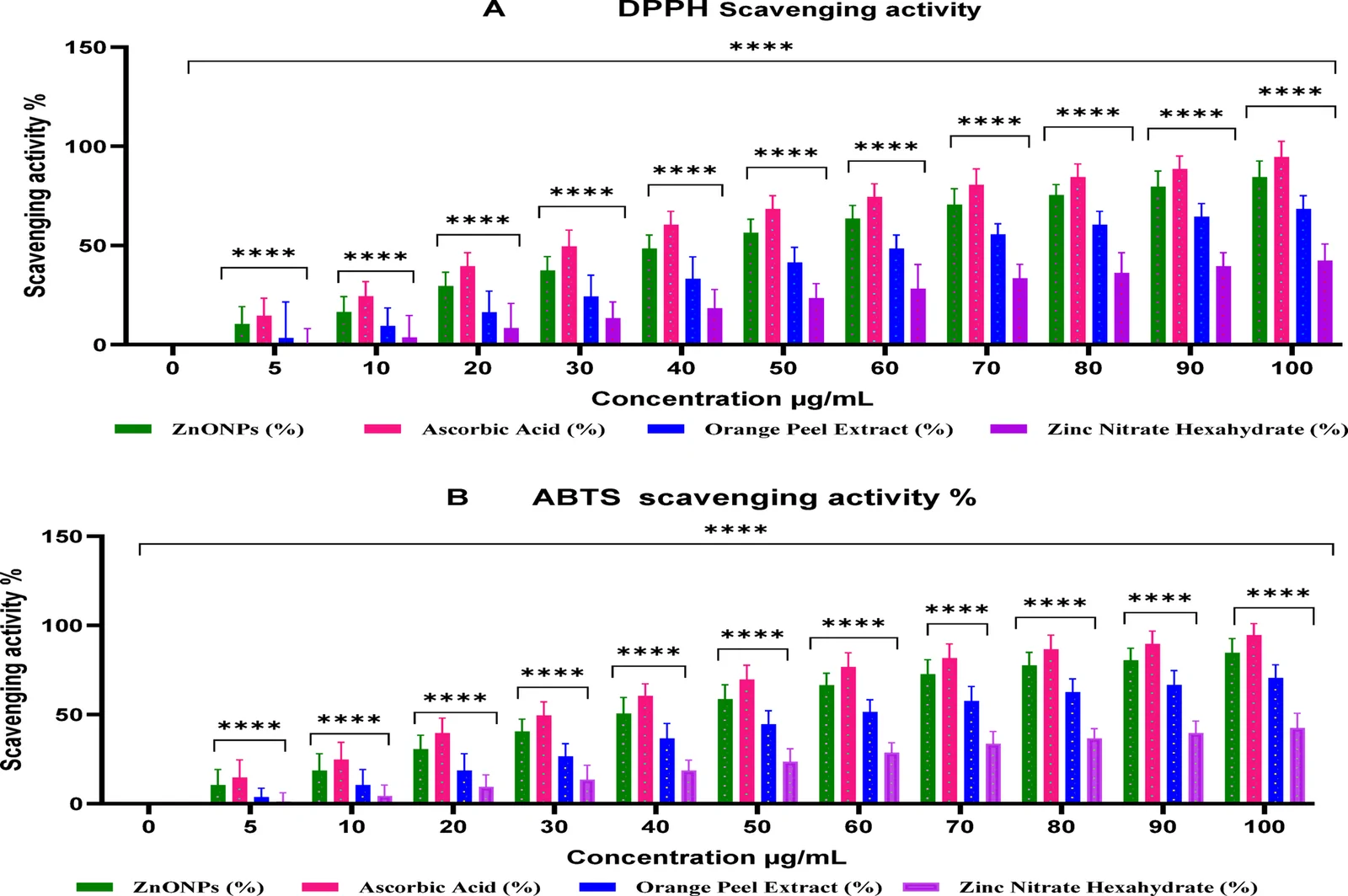
Antioxidant activities of ZnONPs, orange peel extract, and zinc nitrate hexahydrate compared with ascorbic acid. (**A**) DPPH scavenging activity (%), (**B**) ABTS⁺ scavenging activity (%). Data are expressed as mean ± SD (n = 3). Different lowercase letters indicate significant differences among treatments (one-way ANOVA followed by Tukey’s HSD test, *p* < 0.05).

The DPPH assay demonstrated a concentration-dependent increase in radical scavenging activity for all tested samples (Fig. 4A). Ascorbic acid exhibited the strongest antioxidant activity across all concentrations, increasing from approximately 18% scavenging at 5 µg/mL to nearly 100% at 100 µg/mL. ZnONPs showed substantial antioxidant potential, with scavenging activity increasing from approximately 12% at 5 µg/mL to about 90% at 100 µg/mL. In comparison, orange peel extract displayed moderate activity, reaching approximately 72% inhibition at the highest concentration tested. Zinc nitrate hexahydrate exhibited the lowest scavenging capacity throughout the concentration range, with activity increasing only from approximately 5% to 45%. Statistical analysis revealed significant differences among the tested treatments at each concentration (p < 0.0001). ZnONPs exhibited significantly higher DPPH scavenging activity than both orange peel extract and zinc nitrate hexahydrate at all tested concentrations, although their activity remained significantly lower than that of the ascorbic acid standard. The overall dose-dependent trend was highly significant, confirming the concentration-dependent antioxidant behavior of ZnONPs.

Similarly, the ABTS assay showed a marked concentration-dependent increase in radical scavenging activity for all treatments (Fig. 4B). Ascorbic acid again exhibited the highest activity, increasing from approximately 20% at 5 µg/mL to nearly 100% at 100 µg/mL. ZnONPs demonstrated strong ABTS scavenging activity, rising from approximately 10% at 5 µg/mL to around 92% at 100 µg/mL. Orange peel extract displayed intermediate activity, reaching approximately 75% scavenging at the highest concentration, whereas zinc nitrate hexahydrate showed the weakest activity, attaining only about 45% inhibition at 100 µg/mL.

Statistical comparisons indicated highly significant differences (p < 0.0001) among treatments at all concentrations. The antioxidant activity of ZnONPs was significantly greater than that of the orange peel extract and zinc nitrate hexahydrate, suggesting that nanoparticle synthesis enhanced the free-radical scavenging potential of the plant extract. The progressive increase in scavenging activity with increasing concentration further confirmed a significant dose–response relationship in the ABTS assay.

The IC₅₀ values obtained from the DPPH and ABTS assays indicated that ascorbic acid possessed the highest antioxidant potency (IC₅₀ ≈ 27–28 µg/mL), followed by ZnONPs (≈ 38–40 µg/mL) and orange peel extract (≈ 60–62 µg/mL). Zinc nitrate hexahydrate exhibited weak antioxidant activity, with IC₅₀ values exceeding 100 µg/mL. These results confirm that biosynthesized ZnONPs display markedly improved free-radical scavenging capacity compared with the crude plant extract.

Orange peel extract contains various bioactive compounds, including flavonoids, phenolic acids, ascorbic acid, and carotenoids, which contribute to antioxidant activity through radical scavenging and reducing mechanisms^1^. During nanoparticle synthesis, these phytochemicals likely functioned as reducing and stabilizing agents, enhancing the surface reactivity and antioxidant behavior of ZnONPs.

The stronger antioxidant activity of ZnONPs relative to zinc nitrate hexahydrate confirms that nanoparticle formation significantly enhances bioactivity beyond that of the precursor metal salt. Similar improvements in antioxidant potential have been reported for green-synthesized ZnO nanoparticles prepared using plant extracts rich in phenolic compounds^58,63^.

Previous studies have similarly reported that green synthesized ZnONPs exhibit strong antioxidant activities due to surface-bound bioactive compounds and nanoscale particle properties^58,63^. The ABTS assay produced slightly higher scavenging percentages than the DPPH assay for both ZnONPs and ascorbic acid, suggesting that ABTS radicals are more susceptible to reduction by the synthesized nanoparticles. This observation agrees with previous reports demonstrating enhanced ABTS scavenging capacity of phytochemical-mediated ZnONPs owing to their improved electron transfer ability and higher surface reactivity^58,64^. Overall, the findings demonstrate that biosynthesized ZnONPs possess considerable antioxidant activity, although slightly lower than that of ascorbic acid. The strong radical scavenging capacity of ZnONPs highlights their potential application as natural antioxidant agents in biomedical, pharmaceutical, and food preservation applications.

### In vitro*,* anticancer activity evaluation of biosynthesized zinc oxide nanoparticles

**Fig. 5:**
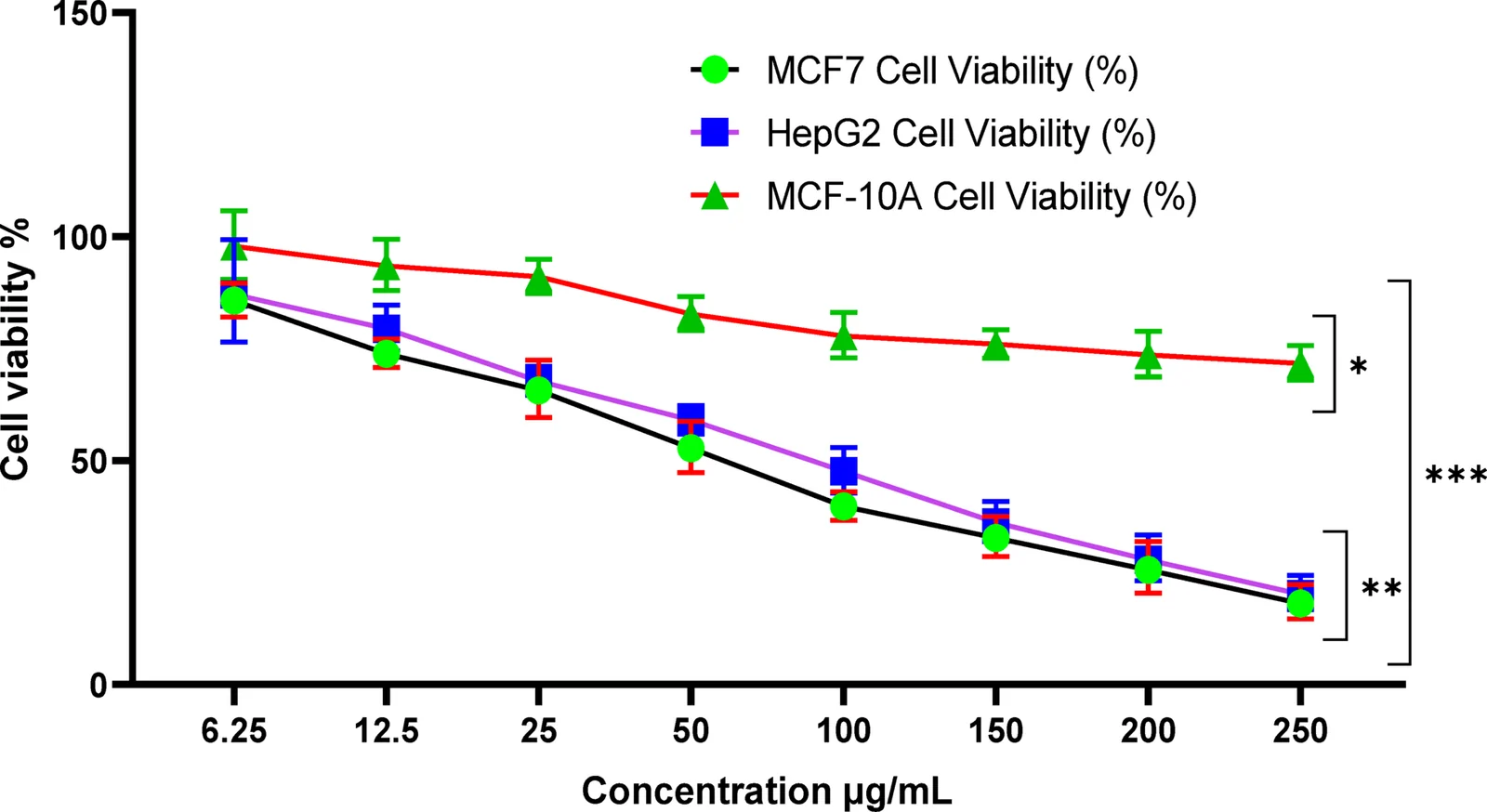
Cytotoxicity of biosynthesized ZnONPs (6.25–250 µg/mL) on the viability of MCF-7, HepG2, and MCF-10A cells after 24 h of treatment, determined by the MTT assay. Data are presented as mean ± SD (n = 3). Biosynthesized ZnONPs significantly reduced the viability of MCF-7 and HepG2 cells in a dose-dependent manner, while exhibiting lower cytotoxicity toward MCF-10A cells.

The anticancer activity of the biosynthesized ZnONPs was initially evaluated against human breast adenocarcinoma (MCF-7), hepatocellular carcinoma (HepG2), and normal breast epithelial (MCF-10A) cells using the MTT assay after 24 h of exposure to concentrations ranging from 6.25 to 250 µg/mL (Fig. 5). MCF-10A cells were included to assess the cytotoxic selectivity of ZnONPs toward normal cells. Exposure to ZnONPs reduced MCF-7 cell viability from 85.4 ± 1.5% at 6.25 µg/mL to 17.8 ± 1.3% at 250 µg/mL, whereas HepG2 cell viability declined from 86.2 ± 1.6% to 19.9 ± 1.5% over the same concentration range. MCF-7 cells consistently exhibited greater sensitivity than HepG2 cells. In contrast, MCF-10A cells maintained relatively high viability, decreasing only from 97.3 ± 2.1% to 71.4 ± 1.8%, confirming the preferential cytotoxicity of ZnONPs toward malignant cells.

Statistical analysis revealed significant differences between cancerous and normal cell lines at all tested concentrations, with significance increasing from p < 0.05 at the lowest dose to p < 0.0001 at higher concentrations. ZnONPs also produced significantly greater growth inhibition than orange peel extract or zinc nitrate hexahydrate (p < 0.05), confirming that the observed anticancer activity was primarily attributable to nanoparticle formation.

Dose–response analysis yielded IC₅₀ values of 58.98 µg/mL for MCF-7 and 87.93 µg/mL for HepG2 cells, whereas the IC₅₀ for MCF-10A cells exceeded 250 µg/mL. Corresponding selectivity indices (SI > 4.24 for MCF-7 and > 2.84 for HepG2) indicate a favorable therapeutic window and demonstrate that orange peel-mediated ZnONPs selectively target cancer cells while largely sparing normal breast epithelial cells.

The superior biological performance of green-synthesized ZnONPs using orange peel extract may be further explained by phytochemical-mediated reduction, stabilization, and surface functionalization of nanoparticles^64^. Bioactive compounds present in citrus peel, including flavonoids, phenolics, and polymethoxyflavones, act as both reducing and capping agents, enhancing nanoparticle stability, dispersibility, and cellular uptake. Such green-mediated nanostructures often demonstrate improved biocompatibility and therapeutic efficiency compared with chemically synthesized counterparts^1,13^. Although orange peel extract alone exhibited moderate cytotoxicity, likely due to constituents such as hesperidin, naringin, gallic acid, limonoids, carotenoids, and polymethoxyflavones, its anticancer effects remain primarily associated with antioxidant activity, inhibition of proliferation, and apoptosis induction in breast cancer cells^65,66^.

The enhanced cytotoxic activity of ZnONPs can be attributed to multiple interconnected mechanisms, including increased intracellular ROS generation, mitochondrial dysfunction, oxidative stress–mediated apoptosis, membrane disruption, and DNA damage. Owing to their high surface area-to-volume ratio, nanoparticles exhibit efficient cellular internalization and strong interactions with intracellular biomolecules, thereby amplifying their biological effects^13^. Numerous studies have reported that ZnONPs selectively induce apoptosis in cancer cells through caspase activation and ROS-dependent signaling^58,67,68^.

In contrast, ZnONPs demonstrated significantly stronger cytotoxicity, suggesting a synergistic enhancement between zinc oxide nanostructures and phytochemical surface moieties. The weak activity of zinc nitrate hexahydrate further confirms that the precursor salt itself contributes minimally to cytotoxic effects, indicating that nanoparticle formation is the primary driver of anticancer activity. The observed reduction in MCF-7 cell viability is strongly associated with ROS-mediated mechanisms, where ZnONPs induce oxidative stress, membrane damage, mitochondrial dysfunction, and activation of caspase-dependent apoptotic pathways^69,70^. The IC₅₀ values obtained in this study are consistent with previously reported biosynthesized ZnO nanoparticles against breast cancer cell lines^70,71^. Furthermore, Eshkelani et al.^67^ demonstrated that ZnO nanoparticle conjugates enhance apoptotic activity via mitochondrial dysfunction and oxidative stress amplification.

### Cellular uptake and intracellular localization of biosynthesized ZnONPs

**Fig. 6:**
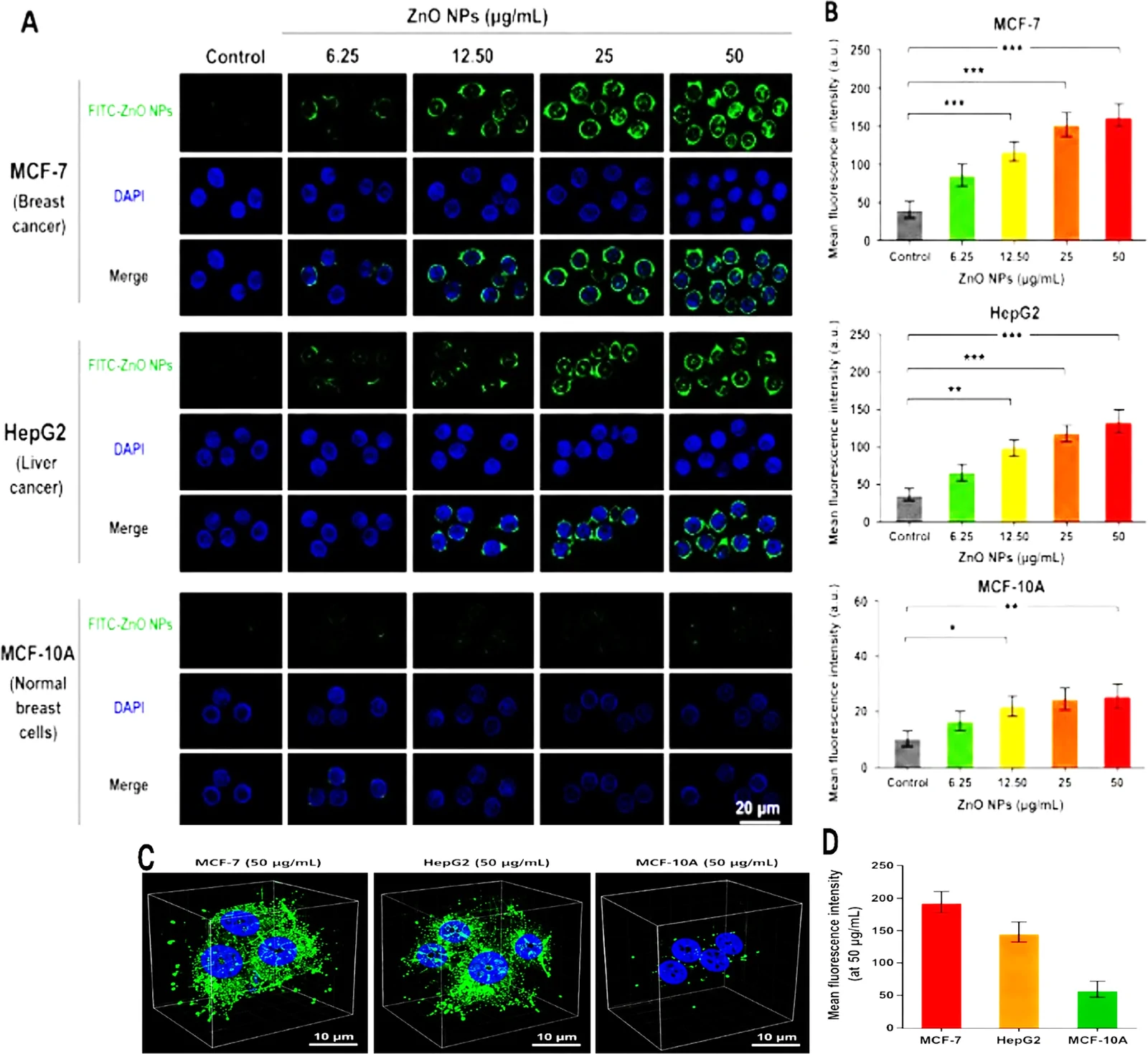
Cellular uptake and intracellular localization of FITC-labeled ZnONPs in MCF-7, HepG2, and MCF-10A cells. (**A**) Confocal images showing concentration-dependent uptake of FITC-ZnONPs (6.25–50 µg/mL; green); nuclei were stained with DAPI (blue) (scale bar = 20 µm). (**B**) Quantification of fluorescence intensity demonstrating higher uptake in MCF-7 and HepG2 cells than in MCF-10A cells. (**C**) Three-dimensional confocal reconstruction of intracellular ZnONP localization at 50 µg/mL (scale bar = 10 µm). (**D**) Quantitative analysis confirming preferential accumulation of ZnONPs in cancer cells. Data are presented as mean ± SD (n = 3); *p < 0.05, **p < 0.01, and ***p < 0.001 versus control.

To elucidate the mechanism underlying the selective anticancer activity of the biosynthesized ZnONPs, cellular uptake was investigated in MCF-7, HepG2, and normal MCF-10A cells using FITC labeling combined with two-dimensional and three-dimensional confocal laser scanning microscopy (CLSM) (Figs. 6A–D). Confocal images revealed a concentration-dependent increase in intracellular fluorescence following treatment with ZnONPs (6.25–50 µg/mL) in all cell lines. Notably, MCF-7 cells exhibited the strongest fluorescence intensity, followed by HepG2 cells, whereas MCF-10A cells displayed only weak fluorescence signals even at the highest concentration, indicating substantially lower nanoparticle uptake.

Merged FITC/DAPI images confirmed that ZnONPs were efficiently internalized into the cytoplasm rather than merely adsorbing onto the cell membrane. Quantitative analysis demonstrated a significant dose-dependent increase in nanoparticle uptake in MCF-7 and HepG2 cells compared with untreated controls (*p* < 0.001). At 50 µg/mL, MCF-7 cells showed the highest mean fluorescence intensity, followed by HepG2 cells, while MCF-10A cells exhibited significantly lower fluorescence (*p* < 0.001). Three-dimensional confocal reconstruction further validated the extensive intracellular distribution of ZnONPs throughout the cytoplasm of cancer cells, whereas only sparse fluorescence was observed in normal cells.

Importantly, the cellular uptake profile closely paralleled the cytotoxicity results obtained from the MTT assay. MCF-7 cells, which exhibited the greatest nanoparticle accumulation, also displayed the highest sensitivity to ZnONPs (IC₅₀ = 58.98 µg/mL), followed by HepG2 cells (IC₅₀ = 87.93 µg/mL), whereas MCF-10A cells remained largely resistant (IC₅₀ > 250 µg/mL). These findings indicate that preferential nanoparticle internalization is a major determinant of the selective antiproliferative activity of ZnONPs.

The enhanced uptake observed in cancer cells may be attributed to their increased metabolic activity, altered membrane properties, and elevated endocytic capacity compared with normal cells^1,2^. Following internalization, ZnONPs are known to accumulate within endosomal and lysosomal compartments, where gradual Zn^2^⁺ release stimulates excessive ROS generation, mitochondrial dysfunction, and activation of intrinsic apoptotic signaling pathways^67–70^. This mechanism is consistent with the significant upregulation of the pro-apoptotic gene *BAX* and suppression of the anti-apoptotic gene *BCL2* observed in the present study.

Furthermore, the superior cellular uptake and tumor selectivity of the biosynthesized ZnONPs may be enhanced by the phytochemical corona derived from orange peel extract. Surface-bound flavonoids, phenolic compounds, and organic acids can improve nanoparticle stability, biocompatibility, and interactions with cancer cell membranes, thereby facilitating intracellular delivery^1,68,69^. Similar findings have been reported for other green-synthesized ZnONPs, which exhibit improved biological performance compared with chemically synthesized nanoparticles.

Collectively, the integration of confocal imaging, quantitative fluorescence analysis, cytotoxicity assays, and apoptosis-related gene expression provides compelling mechanistic evidence that preferential intracellular accumulation is closely associated with the selective anticancer efficacy of orange peel-mediated ZnONPs. These results highlight the potential of citrus waste-derived ZnONPs as promising multifunctional nanotherapeutics for targeted cancer treatment.

### Effects of ZnONPs on apoptosis-related gene expression

**Fig. 7:**
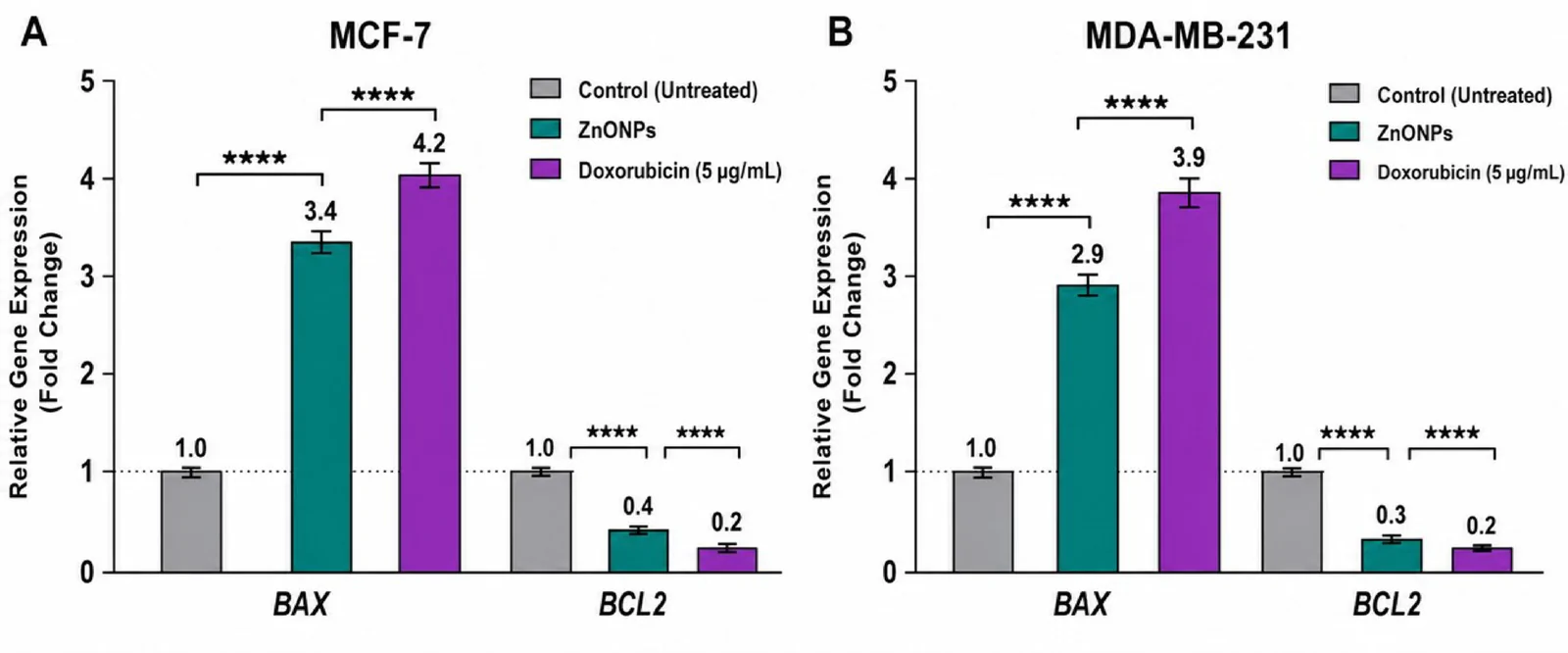
Effect of ZnONPs and doxorubicin (5 µg/mL, positive control) on *BAX* and *BCL2* gene expression in MCF-7 (**A**) and MDA-MB-231 (**B**) cells. ZnONPs significantly upregulated *BAX* and downregulated *BCL2* compared with untreated controls. Data are mean ± SD (n = 3). **** p < 0.0001 vs. control.

Apoptosis is a tightly regulated physiological process that maintains tissue homeostasis through the selective elimination of damaged, dysfunctional, or malignant cells. Dysregulation of apoptotic signaling is a hallmark of cancer progression, contributing to uncontrolled proliferation, metastasis, and therapeutic resistance^1^. Following the demonstration of selective cytotoxicity, the apoptosis study was designed to investigate the molecular mechanism of ZnONP-induced cell death in two biologically distinct breast cancer subtypes: luminal (MCF-7) and triple-negative (MDA-MB-231) as shown in Fig. 7. MCF-10A cells were not included in this analysis because their minimal cytotoxic response (IC₅₀ > 250 µg/mL) indicated limited induction of apoptosis under the tested conditions, whereas the objective of this experiment was to evaluate the pro-apoptotic mechanism in malignant breast cells**.** ZnONPs markedly modulated the expression of apoptosis-related genes in both MCF-7 and MDA-MB-231 cells, indicating activation of the intrinsic mitochondrial apoptotic pathway. In MCF-7 cells, ZnONP treatment significantly increased the expression of the pro-apoptotic gene *BAX* to approximately 3.4-fold while reducing the anti-apoptotic *BCL2* expression to approximately 0.4-fold relative to untreated controls. Similarly, in MDA-MB-231 cells, *BAX* expression increased to approximately 2.9-fold, accompanied by a marked reduction in *BCL2* expression to approximately 0.3-fold. Treatment with doxorubicin (5 µg/mL), used as a positive control, produced comparable apoptotic responses, further validating the potent pro-apoptotic activity of the biosynthesized ZnONPs. These transcriptional changes were highly significant (p < 0.0001) and collectively demonstrate that ZnONPs induce apoptosis through modulation of mitochondrial apoptosis-regulatory genes, as illustrated in Fig. 7A and B. Collectively, these findings demonstrate that ZnONPs strongly shifted the apoptotic balance toward a pro-apoptotic phenotype in both breast cancer cell lines. The ratio between *BAX* and *BCL2* is a critical regulator of mitochondrial outer membrane permeabilization and serves as a decisive molecular checkpoint governing cytochrome c release, caspase cascade activation, and subsequent apoptotic cell death^72,73^. Therefore, the marked increase in the *BAX/BCL2* ratio observed following ZnONPs treatment strongly indicates induction of mitochondrial-mediated apoptosis, which is widely recognized as a major mechanism underlying anticancer efficacy^74,75^. The potent apoptogenic activity of ZnONPs may be attributed to multiple interconnected molecular mechanisms. ZnONPs have been extensively reported to induce intracellular reactive oxygen species (ROS) generation, leading to oxidative stress, mitochondrial dysfunction, DNA damage, and activation of intrinsic apoptotic signaling pathways^76^. Excessive ROS accumulation promotes mitochondrial membrane depolarization and facilitates translocation of *BAX* proteins to the mitochondrial membrane, thereby enhancing membrane permeabilization and apoptotic signaling while suppressing *BCL2*-mediated cell survival pathways^77^. The observed suppression of *BCL2* expression in the present study further supports disruption of mitochondrial integrity and increased susceptibility of cancer cells to programmed cell death. In addition to ROS-mediated mechanisms, ZnONPs have been shown to regulate several upstream signaling pathways involved in apoptosis modulation, including p53, MAPK, and PI3K/Akt pathways^78^. Activation of p53 signaling can transcriptionally upregulate *BAX* while repressing anti-apoptotic proteins such as *BCL2*. Similarly, inhibition of the PI3K/Akt survival pathway has been associated with reduced cellular proliferation, suppression of anti-apoptotic signaling, and enhanced apoptotic sensitivity in various cancer models, including breast cancer^79,80^. The significant increase in *BAX* expression accompanied by profound *BCL2* suppression observed in this study is therefore indicative of coordinated modulation of mitochondrial apoptotic signaling pathways by ZnONPs. The consistent apoptotic response observed in both MCF-7 and MDA-MB-231 cells suggests that ZnONPs exert broad-spectrum anticancer activity independent of molecular subtype. Nevertheless, the slightly greater transcriptional response observed in MCF-7 cells may reflect differences in hormonal receptor status, mitochondrial priming, and intracellular redox regulation between the two breast cancer cell lines^81^. MCF-7 cells are estrogen receptor-positive and generally exhibit higher mitochondrial apoptotic sensitivity, whereas MDA-MB-231 cells represent a more aggressive triple-negative phenotype with increased resistance to apoptosis-inducing stimuli. Overall, the present findings demonstrate that ZnONPs effectively induce apoptosis in breast cancer cells through modulation of mitochondrial apoptotic regulators, characterized by significant upregulation of *BAX* and downregulation of *BCL2*. These results highlight the therapeutic potential of ZnONPs as promising nanostructured anticancer agents capable of targeting apoptosis-associated pathways in breast cancer therapy. Compared with ZnONPs synthesized using other *Citrus* species, including *Citrus limon*, *Citrus aurantifolia*, *Citrus paradisi*, and *Citrus reticulata*, the orange (*Citrus sinensis*) peel-mediated ZnONPs developed in the present study demonstrated competitive physicochemical characteristics and multifunctional biological activities while offering distinct sustainability advantages^82–86^. Orange peel is one of the most abundant agro-industrial wastes worldwide and is particularly rich in flavonoids (hesperidin and naringin), phenolic acids, ascorbic acid, citric acid, and terpenoids, which act synergistically as natural reducing, capping, and stabilizing agents during nanoparticle biosynthesis^82,83^. These phytochemical constituents contribute to improved nanoparticle stability, enhanced surface functionalization, and favorable biocompatibility, while eliminating the need for hazardous chemical reagents^84^. Moreover, the conversion of orange peel waste into high-value ZnONPs supports waste valorization and circular bioeconomy principles, providing an environmentally sustainable and cost-effective alternative to conventional synthesis routes and other *Citrus*-based green synthesis strategies^84,86^. Collectively, these advantages position orange peel waste as an excellent biogenic resource for producing multifunctional ZnONPs with significant potential for biomedical and antimicrobial applications.

## Conclusion, study limitations, and future perspectives

### Conclusion

This study demonstrates the successful green synthesis of zinc oxide nanoparticles using orange peel extract as a natural reducing, stabilizing, and capping agent, highlighting a sustainable and eco-friendly nanofabrication strategy. Comprehensive physicochemical characterization by UV–Vis, FTIR, XRD, DLS, SEM, TEM, HR-TEM, and SAED confirmed the formation of highly crystalline ZnONPs with well-defined nanoscale morphology, excellent colloidal stability, and functionalized surfaces. FTIR analysis further verified the involvement of phytochemicals, including phenolics, flavonoids, and polysaccharides, in nanoparticle formation and stabilization.

Biological evaluation demonstrated that the biosynthesized ZnONPs possess potent multifunctional properties, including broad-spectrum antimicrobial, antibiofilm, antioxidant, and selective anticancer activities. Determination of MIC, MBC/MFC, and time–kill kinetics confirmed the concentration- and time-dependent bactericidal and fungicidal effects of ZnONPs, with complete microbial eradication achieved at higher concentrations. Moreover, ZnONPs exhibited pronounced dose-dependent cytotoxicity against MCF-7 and HepG2 cancer cells while exerting minimal toxicity toward normal MCF-10A cells, indicating a favorable therapeutic window. Mechanistic investigations revealed significant induction of apoptosis through upregulation of the pro-apoptotic gene *BAX* and downregulation of the anti-apoptotic gene *BCL2*, supporting activation of the intrinsic mitochondrial apoptotic pathway.

Furthermore, confocal microscopy demonstrated preferential cellular uptake and intracellular accumulation of ZnONPs in cancer cells compared with normal cells, providing mechanistic evidence for their selective cytotoxicity and apoptosis-inducing activity. Overall, these findings underscore the dual advantages of environmental sustainability and biomedical applicability, positioning orange peel-mediated ZnONPs as promising multifunctional nanotherapeutics for combating antimicrobial resistance and cancer.

### Study limitations

Despite the encouraging outcomes, several limitations should be acknowledged. First, the study is primarily based on in vitro assays, which may not fully replicate the complex biological interactions occurring in vivo. Second, variability in the phytochemical composition of orange peel extract due to source, season, and extraction conditions may affect nanoparticle reproducibility and consistency. Third, the precise molecular mechanisms underlying the observed antimicrobial and anticancer activities remain only partially elucidated and require further mechanistic investigation. In addition, Zn^2^⁺ ion release from the biosynthesized ZnONPs under physiological conditions was not quantified because ICP-OES/ICP-MS facilities were unavailable, limiting the ability to distinguish nanoparticle-mediated effects from ionic zinc contributions.

Furthermore, long-term colloidal stability studies, including the evaluation of hydrodynamic size, polydispersity index, and zeta potential under storage conditions and in biologically relevant media, were beyond the scope of the present work and the available facilities. Consequently, the long-term physicochemical stability of the nanoparticles remains to be established and will be addressed in future investigations. Finally, the biodistribution, pharmacokinetics, and systemic toxicity of the biosynthesized ZnONPs were not evaluated and warrant comprehensive in vivo studies.

### Future perspectives

Future research should focus on in vivo validation of the therapeutic efficacy and safety profile of OPE-mediated ZnONPs using appropriate animal models. Advanced mechanistic studies involving omics-based approaches, molecular docking, and pathway analysis are recommended to elucidate the precise molecular targets responsible for their bioactivity. Standardization of plant extract composition and optimization of synthesis parameters will be essential to improve reproducibility and scalability for industrial applications. Furthermore, integration of ZnONPs into drug delivery systems, wound healing formulations, and combination therapies with existing chemotherapeutic agents could significantly enhance their translational potential. Ultimately, clinical translation studies will be required to confirm their applicability as safe and effective nanomedicine candidates.

## Data Availability

All data generated or analyzed during this study are included in this published article.
